# Eukaryotic RNA-guided endonucleases evolved from a unique clade of bacterial enzymes

**DOI:** 10.1093/nar/gkad1053

**Published:** 2023-11-16

**Authors:** Peter H Yoon, Petr Skopintsev, Honglue Shi, LinXing Chen, Benjamin A Adler, Muntathar Al-Shimary, Rory J Craig, Kenneth J Loi, Evan C DeTurk, Zheng Li, Jasmine Amerasekera, Marena Trinidad, Hunter Nisonoff, Kai Chen, Arushi Lahiri, Ron Boger, Steve Jacobsen, Jillian F Banfield, Jennifer A Doudna

**Affiliations:** Department of Molecular and Cell Biology, University of California, Berkeley; Berkeley, CA, USA; Innovative Genomics Institute; University of California, Berkeley, CA, USA; Innovative Genomics Institute; University of California, Berkeley, CA, USA; California Institute for Quantitative Biosciences (QB3), University of California, Berkeley, CA, USA; Innovative Genomics Institute; University of California, Berkeley, CA, USA; Howard Hughes Medical Institute, University of California, Berkeley; Berkeley, CA, USA; Innovative Genomics Institute; University of California, Berkeley, CA, USA; Department of Earth and Planetary Science, University of California, Berkeley, CA, USA; Innovative Genomics Institute; University of California, Berkeley, CA, USA; California Institute for Quantitative Biosciences (QB3), University of California, Berkeley, CA, USA; Department of Molecular and Cell Biology, University of California, Berkeley; Berkeley, CA, USA; Innovative Genomics Institute; University of California, Berkeley, CA, USA; California Institute for Quantitative Biosciences (QB3), University of California, Berkeley, CA, USA; Department of Molecular and Cell Biology, University of California, Berkeley; Berkeley, CA, USA; Innovative Genomics Institute; University of California, Berkeley, CA, USA; Innovative Genomics Institute; University of California, Berkeley, CA, USA; California Institute for Quantitative Biosciences (QB3), University of California, Berkeley, CA, USA; Department of Molecular, Cell and Developmental Biology, University of California, Los Angeles, CA, USA; Department of Human Genetics, University of California, Los Angeles, CA, USA; Innovative Genomics Institute; University of California, Berkeley, CA, USA; Howard Hughes Medical Institute, University of California, Berkeley; Berkeley, CA, USA; Center for Computational Biology, University of California, Berkeley; Berkeley, CA, USA; Department of Molecular and Cell Biology, University of California, Berkeley; Berkeley, CA, USA; Innovative Genomics Institute; University of California, Berkeley, CA, USA; Department of Molecular and Cell Biology, University of California, Berkeley; Berkeley, CA, USA; Innovative Genomics Institute; University of California, Berkeley, CA, USA; Innovative Genomics Institute; University of California, Berkeley, CA, USA; California Institute for Quantitative Biosciences (QB3), University of California, Berkeley, CA, USA; Department of Molecular, Cell and Developmental Biology, University of California, Los Angeles, CA, USA; Howard Hughes Medical Institute, University of California, Los Angeles CA, USA; Innovative Genomics Institute; University of California, Berkeley, CA, USA; Department of Earth and Planetary Science, University of California, Berkeley, CA, USA; Department of Molecular and Cell Biology, University of California, Berkeley; Berkeley, CA, USA; Innovative Genomics Institute; University of California, Berkeley, CA, USA; California Institute for Quantitative Biosciences (QB3), University of California, Berkeley, CA, USA; Howard Hughes Medical Institute, University of California, Berkeley; Berkeley, CA, USA; Gladstone Institutes; San Francisco, CA, USA; Gladstone-UCSF Institute of Genomic Immunology; San Francisco, CA, USA; Molecular Biophysics and Integrated Bioimaging Division, Lawrence Berkeley National Laboratory; Berkeley, CA, USA; Department of Chemistry, University of California, Berkeley; Berkeley, CA, USA

## Abstract

RNA-guided endonucleases form the crux of diverse biological processes and technologies, including adaptive immunity, transposition, and genome editing. Some of these enzymes are components of insertion sequences (IS) in the IS200/IS605 and IS607 transposon families. Both IS families encode a TnpA transposase and a TnpB nuclease, an RNA-guided enzyme ancestral to CRISPR-Cas12s. In eukaryotes, TnpB homologs occur as two distinct types, Fanzor1s and Fanzor2s. We analyzed the evolutionary relationships between prokaryotic TnpBs and eukaryotic Fanzors, which revealed that both Fanzor1s and Fanzor2s stem from a single lineage of IS607 TnpBs with unusual active site arrangement. The widespread nature of Fanzors implies that the properties of this particular lineage of IS607 TnpBs were particularly suited to adaptation in eukaryotes. Biochemical analysis of an IS607 TnpB and Fanzor1s revealed common strategies employed by TnpBs and Fanzors to co-evolve with their cognate transposases. Collectively, our results provide a new model of sequential evolution from IS607 TnpBs to Fanzor2s, and Fanzor2s to Fanzor1s that details how genes of prokaryotic origin evolve to give rise to new protein families in eukaryotes.

## Graphical Abstract

**Graphical Abstract:**
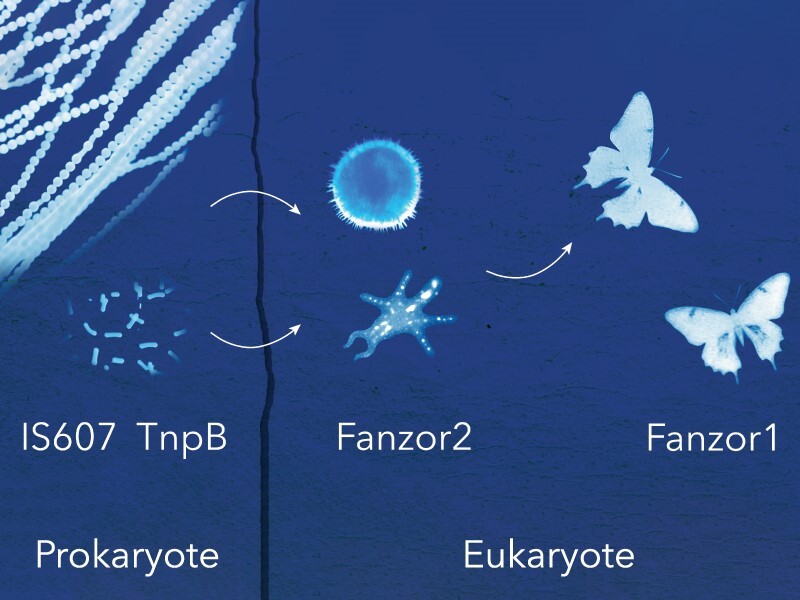

## Introduction

**Figure 1.**
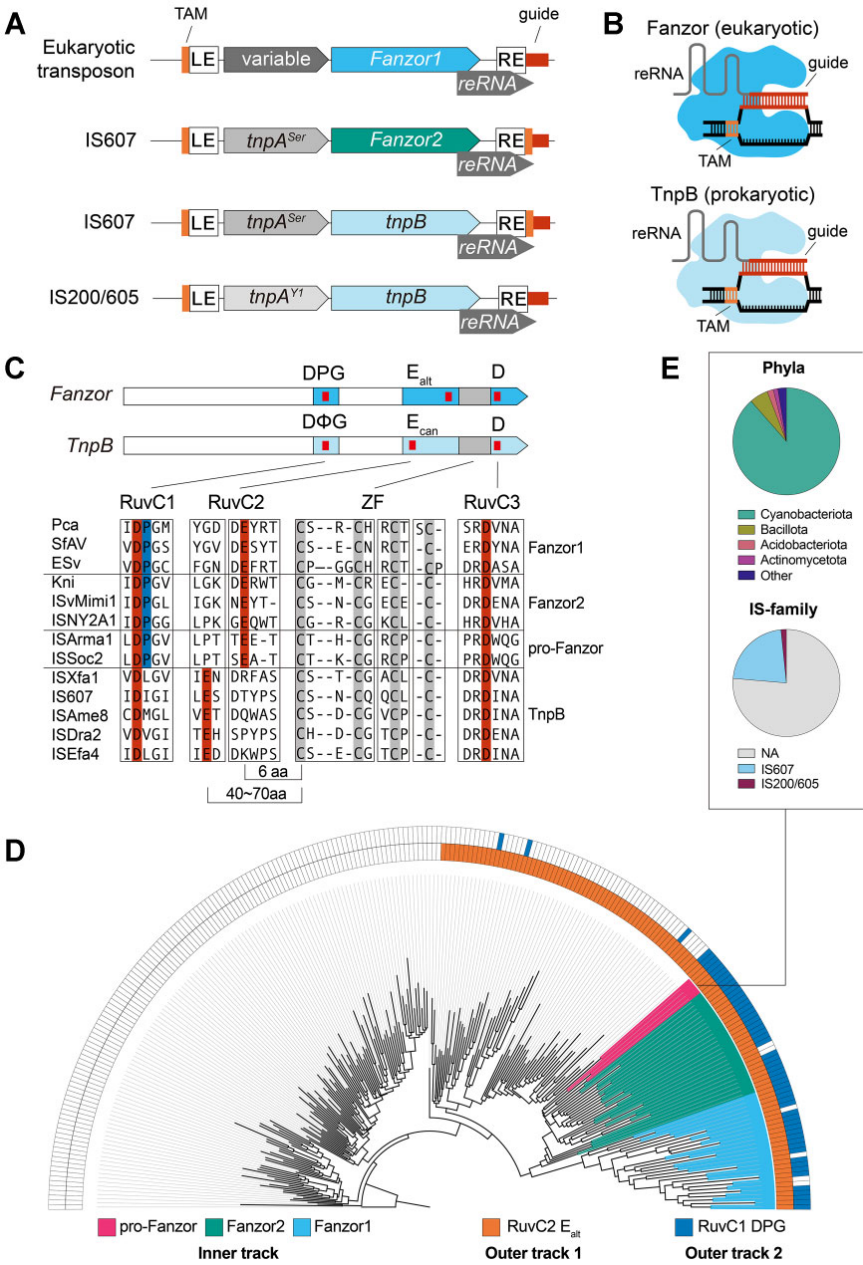
Fanzors and a unique clade of TnpBs share unusual active site signatures. (**A**) Depiction of loci architecture of Fanzor and TnpB containing transposons. LE and RE correspond to left-end and right-end transposon boundaries. The transposon associated motif (TAM) and guide region are highlighted in orange and red, respectively. (**B**) Illustration of Fanzor/TnpB complexed with their right-end RNAs (reRNAs) restricting DNA. Fanzors/TnpBs recognize DNA containing both a TAM and sequence complementarity to the guide region of the reRNA. (**C**) Fanzors and TnpBs with RuvC (blue) and Zinc-Finger (grey) domain annotations. Red ticks indicate locations of D-E-D RuvC triad residues. Fanzors and TnpBs are differentiated by the DPG and DΦG, and E_alt_ and E_can_ signatures in the RuvC domain, respectively. Multiple Sequence Alignment encompassing RuvC and Zinc-Finger domains of Fanzors and TnpBs is shown. (**D**) Maximum likelihood tree of TnpBs and Fanzors annotated by the RuvC features. See legend for the annotations depicted in the track indicated below. (**E**) Pie charts detailing phyla and IS family classification of the prokaryotic group of TnpBs closely related to Fanzor2s (pro-Fanzors) in (D).

Transposons in IS200/605 and IS607 families occur widely in prokaryotes. Both families encode proteins TnpA and TnpB, as well as a non-coding RNA called right-end RNA (reRNA) or ωRNA (Figure 1A) (1,2). Despite these shared features, the TnpA transposases of the two families are unrelated (Figure 1A). IS200/605 transposons encode a Y1-transposase (TnpA^Y1^) of the HUH enzyme superfamily that mediates ‘peel-and-paste’ transposition of single stranded DNA (3). In contrast, IS607 transposons encode a serine recombinase (TnpA^Ser^) that inserts double-stranded DNA into short dinucleotide motifs (4,5). Nevertheless, the two families of transposons share the TnpB gene, the ancestor of type V CRISPR nucleases (Cas12). Analogous to Cas12 and its CRISPR RNA, TnpB and its reRNA mediates RNA-guided DNA target recognition and cleavage (Figure 1B) (1,2).

Unlike Cas12s, the prevalence of TnpB-family homologs extends beyond prokaryotes (3,6,7). TnpBs share homology with eukaryotic Fanzor proteins found in metazoans, fungi and protists, as well as giant eukaryotic viruses (8). Like TnpBs, Fanzors occur within transposons (Figure 1A). However, unlike TnpBs, Fanzors are associated with a wide range of transposon families (Figure 1A) (7,8). Although representative Fanzor proteins have recently been shown to be RNA-guided DNA endonucleases (Figure 1B) (6,7), little is known about their biological function or evolutionary origins.

Fanzor proteins are categorized into two types: Fanzor2s that closely resemble TnpBs and occur in IS607 transposons, and Fanzor1s that are divergent from TnpBs and found in various eukaryotic transposon families (7,8). Transposases associated with Fanzor1s include, but are not limited to, DDE transposases such as hAT or Mariners, Crypton tyrosine-recombinases, and Helitron HUH transposases (7,8). How Fanzors evolved from TnpBs to become widespread and divergent in eukaryotes is poorly understood, although horizontal gene transfer (HGT) has been implicated (6–9).

Here, we show that an unusual group of TnpBs with derived nuclease active site signatures gave rise to Fanzors. Phylogenetic analysis reveals that Fanzors represent a single lineage of proteins originating from a narrow clade of prokaryotic TnpBs. We report IS607 TnpBs primarily found in cyanobacteria as the putative ancestors of Fanzors, and also identify evolutionary intermediates between Fanzor1s and Fanzor2s that bridge their gap. Functional and comparative analysis of IS607 TnpB reRNA and Fanzor reRNA suggest that TnpBs and Fanzors use similar strategies to co-evolve with new transposases. Collectively, our results provide a model that explains how prokaryotic TnpBs evolved and diversified in eukaryotes to become Fanzors. These findings suggest that beyond the ability to catalyze RNA-guided DNA cutting, only one narrow and unique clade of TnpB-family homologs have properties that enabled adaptation in eukaryotic systems.

## Materials and methods

### Metagenomic mining of TnpB-family homologs

The ggKbase platform (https://ggkbase.berkeley.edu/) is a combination of analysis tools for metagenomic datasets. All the protein sequences contained in the database were queried against the Pfam HMM databases of the three TnpB domains: ‘OrfB_IS605’ (pfam01385), ‘HTH_OrfB_IS605’ (pfam12323), ‘OrfB_Zn_ribbon’ (pfam07282) using hmmsearch (HMMER 3.3 (Nov 2019)) with the parameter of ‘–cut_nc’ (10,11). Hits to at least one of three domains were identified as TnpB-family homologs. A total of >140 000 TnpB candidates with a length range of 350–550aa was identified. This master set was further reduced by clustering based on pairwise sequence similarity using MMSeqs2 (12), from which we selected 1078 representative sequences for further analysis (Supplementary Table S1).

### Bioinformatic identification of Fanzor proteins and curation of Fanzor dataset

Non-redundant eukaryotic protein sequences were downloaded from NCBI (November 2022), resulting in 96 197 316 unique sequences. To find putative Fanzor proteins, three HMM models were employed. First, an HMM of the ‘OrfB_Zn_ribbon' (pfam07282) domain was used to search the downloaded set of eukaryotic protein sequences using hmmsearch (HMMER 3.3 (November 2019)) (10). To filter these hits further we built HMMs on two conserved sub-regions of the RuvC domain (RuvC 2 and 3). HMMs of these sub-regions were built by aligning 69 seemingly intact Fanzor sequences (8) that were manually selected based on ColabFold structural prediction (13). Two conserved regions corresponding to RuvC2 and 3 subdomains were manually identified in the alignment passed to hmmbuild (HMMER 3.3 (November 2019)) (10). Sequences that matched the OrfB_Zn_ribbon domain were subsequently searched against the two HMMs (RuvC2 and RuvC3) using hmmsearch. Sequences that contained hits against both of these domains were considered putative Fanzors. Next, we extracted the OrfB_Zn_ribbon domain as well as the RuvC2 and 3 subdomains from the hits using hmmalign (HMMER 3.3 (November 2019)) (10). The extracted regions were used to augment the original alignments of the OrfB_Zn_ribbon and the two RuvC sub-regions by adding new hits to the original alignment using hmmalign. The new alignment was then used to build a new profile HMM using default hmmbuild parameters. New HMMs were built from these augmented alignments and another round of search was conducted as described above. We continued this process for a total of five rounds, which yielded ∼2500 putative Fanzor sequences (Supplementary Table S2). We next realigned these sequences using ClustalW (14), and filtered for sequences that failed to align at highly conserved residues. We next identified sequence clusters with CLANS (15), and further enriched for hits using BLASTp (16). Finally, the accumulated Fanzor sequences were submitted to ColabFold (13), and the resulting models that appeared to be structurally intact (had complete wedge domain assembly and intact RuvC (32)) were selected as the set of ∼350 different Fanzors used for phylogenetic and structural analyses (Supplementary Table S3).

### Analysis of Fanzor2 hits with the canonical RuvC architecture

Previous studies (6,7) have reported select Fanzor2 sequences that lack the derived RuvC2 feature found in Fanzors. These sequences instead have a typical RuvC2 organization found in most TnpBs. In our mining of Fanzor proteins, we also recovered such hits. To assess the possibility that these sequences are not actually Fanzor sequences (and of non-eukaryotic origin) we combined our hits with those reported in (6), filtered for structurally intact proteins via structural prediction, and took neighboring ORFs adjacent to the putative Fanzors as BLASTp queries on NCBI using default parameters (https://blast.ncbi.nlm.nih.gov/Blast.cgi) (17). Following that, the fraction of BLASTp subjects that belong to prokaryotes or associated bacteriophages was quantified. For nearly all instances of the resulting subjects, BLASTp returned a predominant fraction of sequences annotated with non-eukaryotic taxons. In addition, a large fraction of contigs were shorter than 10 kb, which suggested low accuracy of taxonomic annotation. Results of this analysis are summarized in Supplementary Table S4.

### Phylogenetic analysis of TnpBs/Fanzors

To generate the phylogenetic trees, we began by using MMSeq2s (12) to generate a reduced set of sequences to be aligned using ClustalW (14). Sequences that were poorly aligned at the RuvC subdomains were discarded. Following filtering, regions corresponding to the RuvC domain of TnpBs/Fanzors were extracted. Poorly aligned columns from this alignment were filtered using TrimAl (-gt 0.9) (18). This highly stringent method removed gapped regions caused by insertions experienced in Fanzor1s, and retained only the most conserved regions in the RuvC domain. Based on DALI pairwise structural comparisons (http://ekhidna2.biocenter.helsinki.fi/dali/), the resulting alignment only included regions structurally conserved between TnpBs and Fanzors (19). Approximately maximum likelihood phylogenetic trees were generated using FastTree with default parameters (20). For all trees shown in the study, both JTT and WAG models yielded consistent topologies, which we also found to be consistent across different sets of sequences and alignments. Finally, to generate representative trees in the main figure panel, we used Treemmer to reduce the number of leaves to 250 (21). The resulting tree was imported to Interactive Tree of Life (iTOL) for annotation (22).

### Identification and classification of Fanzor related TnpB sequences in prokaryotes

The Max Planck Institute Bioinformatic Toolkit (MPI) PatternSearch tool (https://toolkit.tuebingen.mpg.de/tools/patsearch) was used to identify TnpB sequences on NCBI NR that shared RuvC1/2 sequence features with Fanzors (23). nr_bac, nr_arc and nr_euk were searched against using the following Regular Expressions (RegEx) as queries: DPGx(120160)ExxxxxxCxxCx(12,18)CxxCx(4,8)D and D{P}Gx(120160)ExxxxxxCxxCx(12,18)CxxCx(4,8)D. The first RegEx captures sequences with a DPG motif, followed by a E positioned six residues away from the first CxxC Zinc-Finger motif that is followed by a D residue four to eight residues away. This RegEx effectively identifies TnpBs with a DPG motif at RuvC1 that has the rearranged RuvC2 catalytic glutamate residue positioning. The second RegEx is similar to the first, except the DPG motif requirement was replaced by a D{P}G motif, where {P} corresponds to any amino acid but P. We opted to use RegEx over HMM searches for this search as HMMs were too inclusive and were unable to selectively identify TnpB sequences with specific RuvC features. We further filtered these proteins using NCBI Batch CD tool (https://www.ncbi.nlm.nih.gov/Structure/bwrpsb/bwrpsb.cgi) at default parameters, retaining only hits to 'guided_TnpB' (NF040570), 'guided_TnpB superfamily' (cl45887), 'HTH_OrfB_IS605 superfamily' (cl13722), 'OrfB_Zn_ribbon' (pfam07282), 'OrfB_Zn_ribbon superfamily' (cl37560), 'InsQ superfamily' (cl34004), 'InsQ' (COG0675), 'IS200_TnpB' (NF038281), 'IS200_TnpB' superfamily (cl45733), 'OrfB_IS605' (pfam01385), 'OrfB_IS605' superfamily (cl44414). Overall, this search method recovered 3552 sequences (Supplementary Table S5).

To further annotate the RegEx identified TnpB/Fanzor candidates as being either IS200/605- or IS607-associated, we extracted the amino acid sequences of the immediately neighboring genes based on their NCBI accession ID using a custom Python script. We then examined their PFAMs using NCBI Batch CD tool to determine whether they encoded for IS200/605 or IS607 TnpAs (24). Specifically, we filtered for IS200/605 hits using ‘Y1_Tnp’ (pfam01797), ‘Y1_Tnp_superfamily’ (cl00848) and transpos_IS200 (NF033573), and IS607 hits using ‘transpos_IS607’ (NF033518), ‘transpos_IS607 superfamily’ (cl41297), ‘Resolvase’ (cl02788), and ‘Recombinase’ (cl06512). The transposase association was used to assign TnpBs into IS200/605 or IS607 families. TnpBs lacking a TnpA partner were not further categorized. This information was used to annotate Supplementary Table S5, and used to generate the pie charts shown in Figure 1E.

### Identification and analysis of prokaryotic Fanzor1-like sequences

We used the MPI HMMER tool (https://toolkit.tuebingen.mpg.de/tools/hmmer) to identify prokaryotic Fanzor1-like sequences (23). Using an alignment of curated Fanzor1 sequences generated using ClustalW (14) as the query against euk_nr, we found that an e-value cut-off of e-10 selectively retained Fanzor1s, but not Fanzor2s. We therefore searched against both nr_arc and nr_bac using an e-value cut-off of e-10. Resulting hits were further enriched through PSI-BLAST searches conducted using NCBI’s webportal (https://blast.ncbi.nlm.nih.gov/Blast.cgi) (17). The search taxa was limited to "bacteria", and default parameters were used. In each round, only hits larger than 500aa were retained. Finally, this list was cross referenced to previously published prokaryotic Fanzor1-like sequences (6), which resulted in a total of 29 prokaryotic Fanzor1-like sequences. We assessed the validity of the taxonomic assignment of these sequences by manually examining their encoding contigs. To this end, we used BLASTp searches with default parameters and used genes neighboring the Fanzor1-like sequences (2 genes upstream and downstream) as queries (16). For every query, we computed the number of prokaryotic and eukaryotic sequences, which we report in Supplementary Table S6.

### Co-conservation analysis of TnpA and TnpB/Fanzor pairs

For the co-conservation analysis of TnpA and TnpB/Fanzor2 pairs, TnpBs annotated as IS607-associated were parsed from the ISFinder database, and pooled with 1) TnpBs that shared RuvC1 and 2 features with Fanzors (pro-Fanzors), and 2) Fanzor2s parsed from the ggKbase and NCBI. A total of 197 TnpBs/pro-Fanzors/Fanzor2s having TnpA co-encoded in the transposon were selected, and organized into clusters using CLANS (15). TnpBs and Fanzors in four CLANS clusters proximal to ISXfa1 and Fanzor2s were selected for further analysis (a total of 158), together with their associated IS607 TnpA. MSA was performed on sets of TnpB/Fanzors and separately on TnpA with ClustalW (14), and the values corresponding to (i) TnpA-TnpA and (ii) TnpB-TnpB (Fanzor2-Fanzor2) similarity in the two ‘% identity’ matrices were obtained. These were used to plot the sequence identity histogram of pairs of TnpA and TnpB/Fanzor between two distinct transposable elements (i1 and i2) in MATLAB.

### Manual transposon curation

DNA-sequences of the Fanzor-encoding transposon and flanking sequences (typically 2kb upstream and downstream of the TnpB) were extracted and searched against their respective genome assemblies, or different genome assemblies of the same organism, using blastn with default parameters. BLAST-hits showing high query coverage (>50%) were extended by 2 kb on each end to capture flanking sequences, and then aligned to the query using MAUVE whole genome alignment to identify transposon boundaries and associated features (Geneious 2023.0.1 (https://www.geneious.com)) (16,25). This analysis revealed that Fanzor2-encoding elements from *Chloropicon primus* and various mollusc species including *Mya arenaria, Spisula solida, Mercenaria mercenaria* and *Batillaria attramentaria* included clear target site duplications and (degenerate) terminal inverted repeats.

### *E*.*coli* transposon-associated motif (TAM) depletion assay

ISXfa1 TnpB and reRNA encoding region of the locus was synthesized (Twist Biosciences) and cloned into a vector (Cam resistance) under the control of a single tetracycline-inducible promoter (TetR/pTet) using Golden Gate Assembly. This plasmid was then used for the *E. coli* TAM depletion assays, and small RNA sequencing. Alternatively, the ISXfa1 TnpB and its reRNA were individually Golden Gate cloned into a vector under two different promoters (TetR/pTet for TnpB and pJ23119 for reRNA) and used *E. coli* plasmid interference assays. The 3′-end of the reRNA were flanked by a HDV ribozyme and the guide region was kept as 20-nt. The target sites with different TAM sequences were Golden Gate cloned into a target plasmid with Amp/Carb resistance.

Electrocompetent *E.coli* DH10β competent cells harboring the TAM library plasmid (Amp/Carb resistance) with six randomized nucleotides (6N) flanking the target site were prepared as follows. The randomized regions were introduced by PCR from a target plasmid and then Golden Gate ligated. The Golden Gate products were then transformed into electrocompetent *E. coli* DH10β competent cells (NEB) using 0.1 mm electroporation cuvettes (Bio-Rad) on a Micropulser electroporator (Bio-Rad). The cells were plated on Luria-Bertani (LB)-Agar plates containing ampicillin (100 μg/ml). Over 100 000 colonies were scraped to ensure appropriate coverage of all possible combinations of the randomized TAM region. The cells were then inoculated 1:100 into a 1 l LB culture with ampicillin (100 μg/ml) at 37°C to OD_600_= 0.6. The cells were then cooled to 4°C, pelleted, and sufficiently washed by ice-cold water and 10% glycerol. The pellets were then resuspended in 2 ml 10% glycerol, aliquoted into 50 μl, and stored in -80°C.

These TAM library containing competent cells were then thawed and transformed with 100 ng of the pSingle (Cam resistance) using 0.1 mm electroporation cuvettes (Bio-Rad) on a Micropulser electroporator (Bio-Rad). The cells were recovered in 1 ml LB culture at 37°C for one hour. After recovery, the culture was inoculated 1:100 into 3 ml LB culture containing chloramphenicol (34 μg/ml) and carbenicillin (100 μg/ml; equivalent to Amp). ISXfa1 expression was induced with 200 nM aTc at 26°C for 24 h. The plasmids that had been propagated were then isolated using the QIAgen Miniprep Kit (Qiagen). Preferentially depleted TAM motifs were identified using amplicon sequencing of the TAM library plasmid. The library was sequenced on an Illumina NovaSeq by The High-Throughput Sequencing Core in the UCLA Broad Stem Cell Research Center, with paired-end 150bp run configuration and an average depth of 500 000 reads per sample. TAM preferences were characterized from FASTQ files using a custom Python script. Briefly, six-nucleotide TAMs were extracted and counted. TAM frequencies were then normalized to the sequencing depth of each sample. Sequence logos were generated with Logomaker (version 0.8) using TAMs with a log_2_ fold-change of five or greater relative to the original plasmid library (26).

### *E*.*coli* plasmid interference assays

Electrocompetent *E. coli* DH10β cells were double-transformed with 100 ng of ISXfa1 TnpB and reRNA encoding plasmids (Cam resistance) and 100 ng of a target plasmid (Amp/Carb resistance) using 0.1 mm electroporation cuvettes (Bio-Rad) on a Micropulser electroporator (Bio-Rad). The cells were recovered in 1 ml LB culture at 37°C for 1 h. After recovery, 5-fold dilution series of the recovery culture were prepared and 5 μl of the dilution were spot-plated on double-antibiotic plates (LB-Agar with 34 μg/ml chloramphenicol, 100 μg/ml carbenicillin, and 2 nM anhydrotetracycline) as well as single-antibiotic control plates (LB-Agar plate with 34 μg/ml chloramphenicol and 2 nM anhydrotetracycline). For plates with no visible colonies, 400 μl of the 1 ml recovery culture were all plated on the double antibiotic plate for improving detection limit. The plasmid interference efficiency was quantified by the normalized colony-forming units (Norm. CFU) which represents the CFU on the double-antibiotic plate (carbenicillin/chloramphenicol) divided by the CFU on the single-antibiotic control plate (chloramphenicol).

### *E*.*coli* reRNA expression

The plasmid containing the native sequence of ISXfa1 TnpB and reRNA under the control of a single tetracycline-inducible promoter (TetR/pTet) was transformed into *E.coli* DH5α competent cells (Thermo Fisher Scientific). A single colony was picked and inoculated in 3 ml LB culture with chloramphenicol (34 μg/ml) and carbenicillin (100 μg/ml) in 37°C overnight as starting culture. The starting culture was then inoculated 1:100 in 3 ml LB culture with chloramphenicol (34 μg/ml), carbenicillin (100 μg/ml) and anhydrotetracycline (200 nM) to induce the RNA expression. Once the OD_600_ reached 1.0, 400 μl of the cells were taken and mixed with 800 μl of RNAprotect Bacteria Reagent (QIAgen). After 15 min, the cells were pelleted by centrifuge, and stored in −80°C after removing all the supernatant until RNA-extraction (See Small RNA sequencing section below for details).

### RNA structure prediction

The secondary structures of reRNA were predicted by RNAfold WebServer with default parameters (http://rna.tbi.univie.ac.at/cgi-bin/RNAWebSuite/RNAfold.cgi) (27). The putative pseudoknot (PK) interactions were manually annotated on the secondary structures, assuming similar interactions formed as in ISDra2 TnpB between five nucleotides flanking the guide region and the apical loop region in Stem–Loop 1 (or Triplex-Loop in ISDra2 TnpB).

### Insect cell experiments

Expi-Sf9 (GIBCO) cells were cultured in Expi-CD medium (GIBCO) in Polycarbonate Erlenmeyer Flasks with a vent cap (Corning). Cultures were kept in a shaking incubator (New Brunswick) at 26°C and 100 RPM with a 50mm shaking diameter. Cells were maintained at densities between 5 × 10^5^ and 5 × 10^6^, with the volume of the liquid culture remaining below 1/5th of the total volume of the flask. Cell viability and density were measured using Trypan-Blue (GIBCO) on a CytoSMART Cell Counter (Corning).

Bacmids were generated by cloning in DNA fragments into a pFASTBAC vector through PCR amplification of the vector and insertion of synthesized gene-fragments (Twist Biosciences) via Golden Gate Assembly, using DH5a cells (ZYMO) and transforming the cloned plasmids into MAX EFFICIENCY DH10BAC (GIBCO) or EmBacY (Geneva Biotech) *E. coli* strains following manufacture protocols. Chemically-transformed cells were allowed to recover for 4 h in 2XYT media, and plated on LB-Agar plates containing kanamycin (50 μg/ml), gentamicin (7 μg/ml), tetracycline (10 μg/ml), IPTG (40 μg/ml) and X-gal (100 μg/ml). After 48 h at 37 C, large white colonies were picked and cultured in liquid culture containing kanamycin (50 μg/ml), gentamicin (7 μg/ml), tetracycline (10 μg/ml). Bacmids were purified by lysing *E. coli* using the ZymoPURE Miniprep kit (ZYMO), followed by ethanol precipitation for isolation of bacmid DNA. Transgene insertion into the bacmid was confirmed via PCR using M13 reverse primer and a primer binding to the transgene.

12.5 μg of freshly prepared bacmid was transfected into a 25 ml culture containing 62.5 × 10^6^ Expi-Sf9 cells using ExpiFectamine™ Sf Transfection Reagent following manufacturer instructions (GIBCO). Supernatant containing P0 baculovirus was harvested 3 days post-infection by using centrifugation to separate from cells, and titered using the baculovirus titering kit (Expression Systems) and Attune NxT Flow Cytometer with an autosampler. P0 virus was used directly to infect a 25 ml culture with 5 × 10^6^ Expi-SF9 cells at an MOI of 5. Cells were harvested 3 days post-infection for downstream analysis.

### Small RNA sequencing

Total RNA was extracted using a one-step hot formamide extraction method for all samples, where cells are incubated in a 18 mM EDTA and 95% formamide solution at 65ºC for 5 min (28). Cell debris was removed by centrifugation, and 2 volumes of RNA binding buffer (ZYMO) was added to each volume of RNA sample. 1 volume of ethanol was then added to the mixture, and loaded to a Zymo-Spin IIC column. Following on-column DNAse treatment, total RNA was eluted using water. ∼200 ng of total RNA was used for rRNA depletion reactions using NEBNext rRNA kit (bacteria) following manufacturer protocols. For eukaryotic samples, custom probes designed using NEBNext Custom rRNA depletion tool were used. The rRNA-depleted RNA was purified using a Zymo-Spin IIC column, treated with T4 PNK using T4 PNK buffer (NEB) at 37ºC for 30 min before spiking in ATP and further incubating. Further end treatment was performed using mRNA Decapping Enzyme (NEB) pre-mixed with its buffer directly into the PNK reaction and incubating at 37ºC for 30 min. End-repaired RNA was purified using a Zymo-Spin IIC column, and used for Collibri small-RNA-seq library preparation reactions following manufacturer protocols (INVITROGEN). The resulting NGS adapter ligated cDNA was then amplified with KAPA HiFi HotStart ReadyMix using NGS indexing primers for 25 cycles (ROCHE). Amplified DNA fragments between 150–500nt were size selected by gel extraction purification following gel-electrophoresis in a 4% SYBR-GOLD E-GEL. Before loading into the MiSeq platform for sequencing (150 bp paired-end reads), library concentrations were quantified using KAPA-QUANT qPCR kit (ROCHE). The paired-end reads were merged using BBMerge using a normal merge rate and then mapped onto the TnpB or Fanzor containing locus using Geneious mapper at Low Sensitivity to identify reRNA boundaries (Geneious 2023.0.1 (https://www.geneious.com)).

### Rearing and virus infection of *S. frugiperda* larvae

Insect hemolymph containing budded *Spodoptera frugiperda* ascovirus 1a (SfAV) vesicles was received as a generous gift from Brian Federici (University of California, Merced). *Spodoptera frugiperda* larvae were grown on an artificial diet (Benzon Research) at room-temperature until third-instar stage, when they were pricked with a needle contaminated with SfAV containing hemolymph. Five days post treatment, infected larvae with opaque hemolymph were selected for RNA isolation. For *in vivo* protein expression experiments using recombinant baculovirus, larvae were infected by injecting with baculovirus containing clarified culture media.

### Western blots

Mortar and pestle homogenized larval samples were prepared in a buffer containing 50 mM Tris–HCl, 500 mM NaCl and 5 mM TCEP on ice. Homogenized larval samples were further sonicated in 1.5 ml Eppendorf tubes at 60% power setting, 30 s on/30 s off for a total sonication time of 5 min at 4°C. Large particulates from this solution were filtered using a funnel layered with Kimwipes prior to centrifugation. After removing debris by centrifugation at 16 000 g for 10 min, protein concentration in the supernatant was measured on a nanodrop spectrophotometer. ∼50 mg protein lysate was used as stock that was diluted 5-fold per serial dilution in each well. Lysate and dilutions were denatured in 1× Laemmli buffer at 95°C for 30 s and resolved by SDS-PAGE on a 8–20% gradient gel (BioRad). Following trimming of the gel, proteins were electro transferred to a PVDF membrane activated in methanol for 1 minute. The membrane was blocked with blocking buffer (PBS/0.05% Tween-20 containing 5% milk) for 1 hr, incubated with primary antibody in blocking buffer for 1 h, washed three times with PBS/0.05% Tween-20 for 5 min each, incubated with secondary antibody conjugate in blocking buffer for 1 h, and washed three times again with PBS/0.05% Tween-20 for 5 min each. Protein bands were visualized using LI-COR Odyssey CLx with Image Studio v5.2 software at 700/800 nm channels.

## Results

### Fanzors belong to an unusual group of TnpB-family homologs

To determine the evolutionary origins of Fanzors, we identified diverse Fanzors and evaluated their protein sequence features (Materials and methods). TnpB-family homologs, which include Fanzors, share a RuvC nuclease domain belonging to the RNaseH-like superfamily. Within the RuvC domain, the D–E–D catalytic triad is split into three discontinuous subdomains (RuvC1-3) (Figure 1C). Additionally, many TnpBs and Fanzors contain a C-terminal Zinc-Finger (ZF) motif that is flanked by the RuvC2 and 3 subdomains (Figure 1C).

Although TnpBs and Fanzors share similar domain organizations, two features are different. First, RuvC1 of TnpBs typically contains a DΦG motif where the catalytic aspartate is followed by a hydrophobic residue (Φ = I, L, F, W, Y and M), and a glycine. In contrast, this motif is near exclusively DPG in Fanzors (Figure 1C). Second, RuvC2 of TnpBs typically contains a catalytic glutamate situated ∼50 residues upstream of the ZF motif (defined as canonical E or E_can_). In contrast, RuvC2 of Fanzors are reorganized, with the glutamate positioned six residues upstream of the ZF motif (defined as alternative E or E_alt_) (7). Although Fanzors with E_can_ have previously been interpreted as novel Fanzor subtypes (6), we found that they appear to be prokaryotic TnpBs that have been mis-annotated as eukaryotic Fanzor2s (Supplementary Table S4 and Materials and methods). The combination of RuvC1 (DPG motif) and RuvC2 (E_alt_) features seen in Fanzors is exceedingly rare and found only in <1% of TnpB-family homologs (Supplementary Table S7), making it the molecular fingerprints of Fanzors.

We next explored the evolutionary relationship of TnpB-family homologs in the context of Fanzor molecular fingerprints. We generated a maximum likelihood phylogenetic tree using ∼800 representative sequences selected from over 140 000 TnpB-family homologs (Materials and methods; Supplementary File 1). We found clear separation between sequences with E_can_ and E_alt_ in RuvC2 (Figure 1D). Those with E_alt_ were further divided into two clades with differing RuvC1 motifs: one with DΦG (Figure 1D), and another with DPG (Figure 1D). The clade with DPG fingerprints include not only Fanzors, but also TnpBs closely related to Fanzor2s (Figure 1D). These TnpBs are mostly cyanobacterial, and co-occur with IS607 TnpAs closely related to Fanzor2 TnpAs (Figure 1E and Supplementary Figures S1 and S2). Our analysis suggests that DPG and E_alt_ motifs in the RuvC domain are specific to a narrow and unusual clade of TnpB-family homologs. This evolutionarily related clade includes Fanzor1s, Fanzor2s, and select TnpBs. From here on, we refer to these cyanobacterial TnpBs as ‘pro-Fanzors’ to highlight their special relationship to Fanzor2s (Supplementary Text 1).

### Fanzor1s likely evolved from ancestral Fanzor2s in eukaryotes

Motivated by the relatedness of pro-Fanzors and Fanzor2s, we decided to further investigate the prokaryotic origins of Fanzors. We focused on Fanzor1s, as previous studies suggest that Fanzor1s and Fanzor2s independently evolved from distinct clades of TnpBs (6,7). To find support for this model, we searched for prokaryotic Fanzor1-like sequences, and cross-referenced them with sequences that formed the basis of this model (6). Of the 29 sequences validated with ClustalW and AF2, we found that they were either from short metagenomic contigs with ambiguous taxonomy (*n* = 24), or incorrectly ascribed to prokaryotic taxa (*n* = 5) (Supplementary Tables S4 and S5). We therefore find no support for the independent evolution of Fanzor1s from prokaryotic Fanzor1-like proteins.

We next evaluated an alternative model where Fanzor1s evolved from Fanzor2s. A defining feature of Fanzor1s is that they are encoded by a wide range of canonical eukaryotic transposon families. In contrast, Fanzor2s were suggested to be only encoded in eukaryotic IS607 transposons (8). As evolutionary intermediates would support a model where Fanzor1s evolved from Fanzor2s, we searched for either Fanzor1s encoded in IS607 transposons, or Fanzor2s encoded in eukaryotic transposons (Materials and methods).

**Figure 2.**
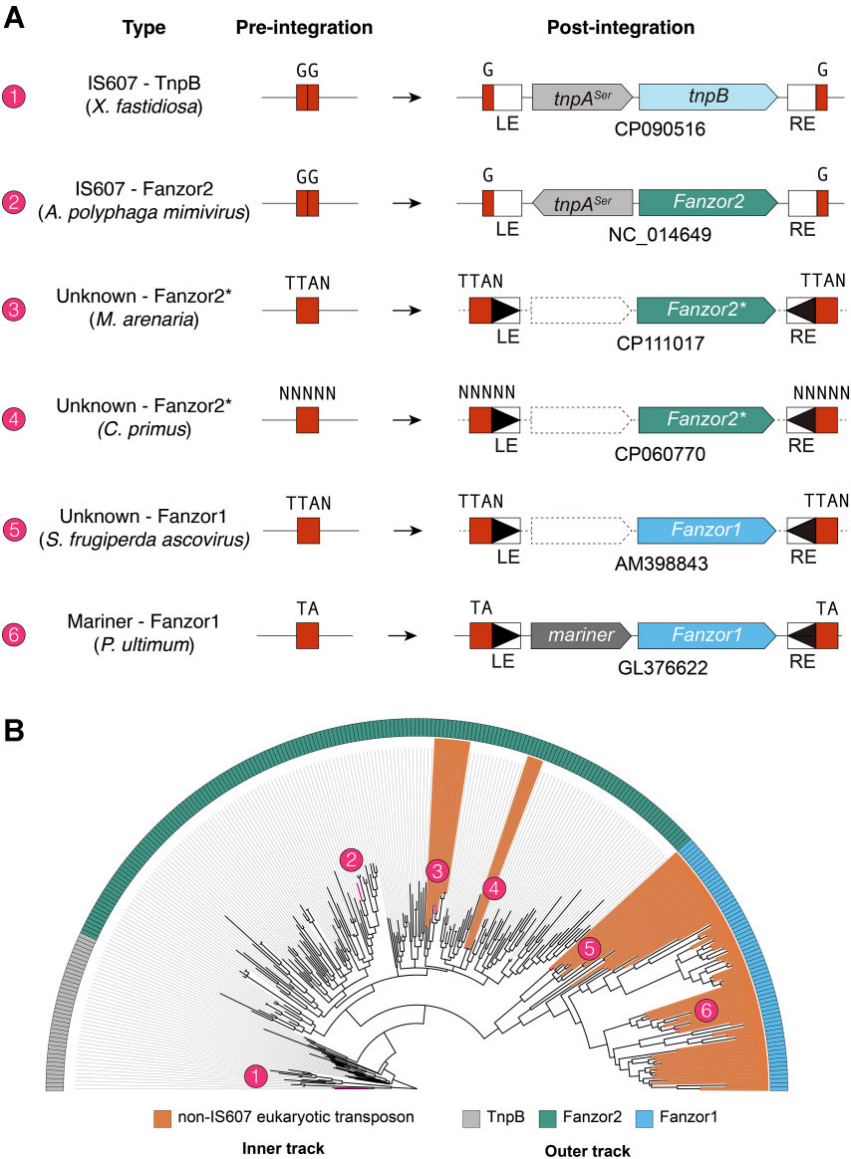
Fanzor2s have been captured by non-IS607 transposons on multiple occasions. (**A**) Depiction of pre-integration and post-integration loci architecture of various TnpB and Fanzor encoding transposons. (**B**) Maximum likelihood phylogenetic tree of TnpBs, Fanzor2s, and Fanzor1s (gray, green and blue in the outer track) annotated based on their transposon associations (inner track). The numbers correspond to transposons and their loci architecture in (A).

Surprisingly, while no Fanzor1s appeared to be encoded in IS607 transposons, some Fanzor2s were clearly encoded by non-IS607 transposons (Figure 2A; Supplementary Figure S3). Specifically, Fanzor2s from the alga *Chloropicon primus* and various mollusc species including *Mercenaria mercenaria* are encoded by cut-and-paste DNA transposons (Figure 2A; Supplementary Figure S3). The terminal inverted repeats (TIRs) and target site duplications (TSDs) suggest that the algal and the mollusc Fanzors are encoded by two distinct transposon families. Consistent with this, phylogenetic analysis suggests that the algal and mollusc Fanzors are distantly related, implying independent capture of distinct IS607 Fanzor2s by eukaryotic transposons (Figure 2B; Supplementary File 2). From here on, we will refer to Fanzor2s encoded in eukaryotic transposons as 'Fanzor2*s' to differentiate them from canonical IS607 encoded Fanzor2s. In all, we propose that Fanzor1s evolved from ancestral Fanzor2s, based on (i) the conserved RuvC signatures and phylogenetic analysis, (ii) the absence of convincing prokaryotic Fanzor1-like proteins, and (iii) the observation of evolutionary intermediates of Fanzor1s and Fanzor2s (Fanzor2s captured by eukaryotic transposons).

### IS607 TnpB adjusts its reRNA boundary to co-function with its transposase

**Figure 3.**
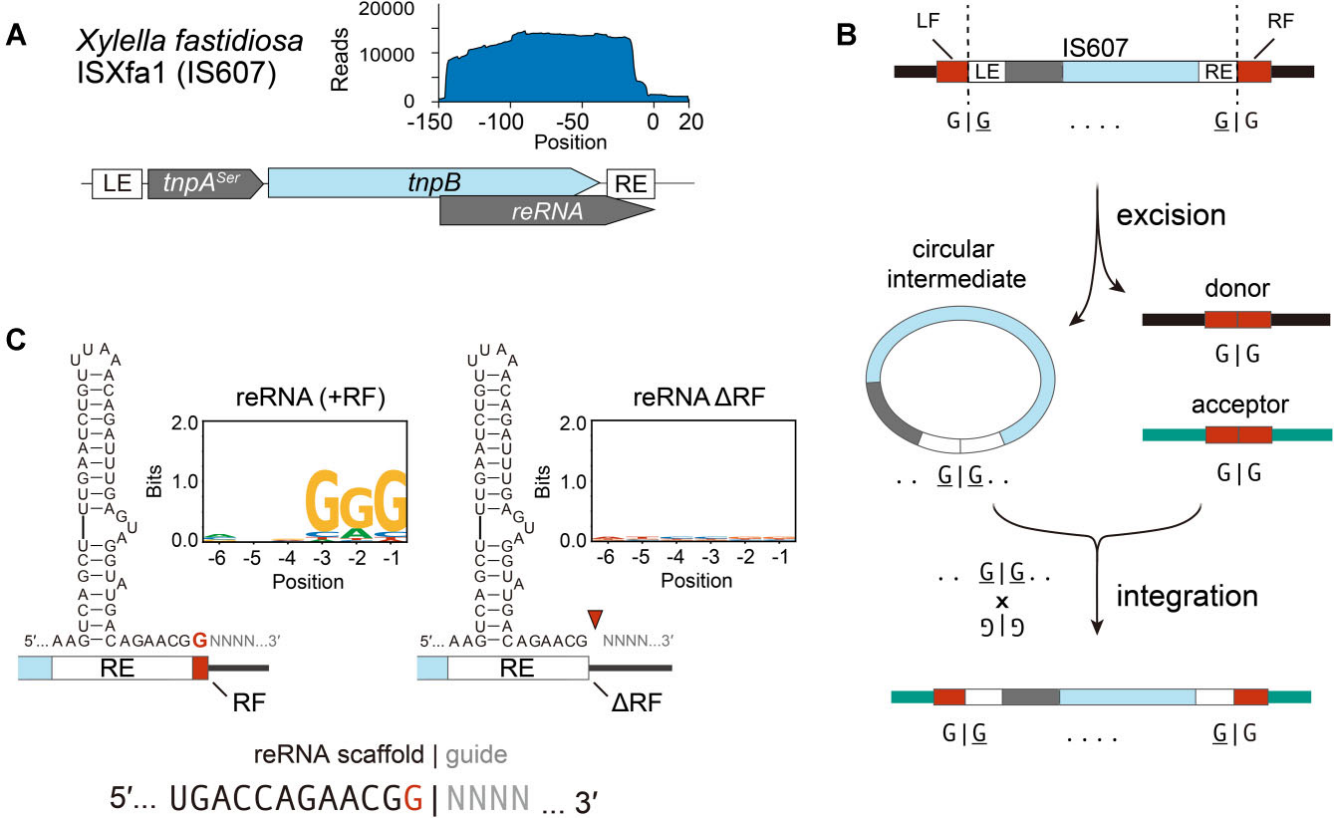
IS607 reRNAs evolved to overcome transposase imposed restrictions. (**A**) Depiction of loci architecture of the ISXfa1 transposon. Inlaid graph shows mapping of small-RNAs to the locus. (**B**) Overview of IS607 transposon life-cycle. LE and RE correspond to left-end and right-end transposon boundaries. LF and RF correspond to left and right-flanking nucleotides encoded in the insertion site. IS607 transposons mobilize as a double-stranded circular DNA intermediate and integrate into the acceptor site, both events happening via crossover between GG dinucleotides at the LE and RE of the transposon, and the donor or acceptor sites. (**C**) Results of TAM depletion assays with variable reRNA boundaries. Wild-type ISXfa1 shows depletion of GGG motif, whereas RF deletion mutant loses activity, demonstrating that the RF nucleotide is part of the reRNA scaffold. This is in contrast to the reRNA architecture of IS200/605-associated TnpBs, whose scaffold ends with the transposon boundaries.

Our results thus far point to an intimate relationship between Fanzors and IS607 TnpBs. The lack of evolutionary connection between Fanzors and IS200/605 TnpBs is notable, as IS200/605 transposons are more abundant than IS607 transposons (29). We hypothesized that features absent in IS200/605 TnpBs, but found in IS607 TnpBs enabled their evolution in eukaryotes. We therefore assayed the biochemical activity of an IS607 TnpB from *Xylella fastidiosa* (ISXfa1) (Figure 3A). Small-RNA sequencing of ISXfa1 TnpB heterologously expressed in *E. coli* revealed a ∼150 nucleotide reRNA that extends beyond the transposon boundary (Figure 3A) (30). This is consistent with IS607 TnpB acting as an RNA-guided endonuclease that uses the reRNA as the guide RNA to cleave a target DNA next to the transposon associated motif (TAM) (30).

Unlike IS200/605 transposons that insert downstream of a short motif (3), IS607 transposons typically insert via recombination between matching dinucleotide motifs (Figure 3B) (4,5). Due to these differences in transposition mechanisms, the first nucleotide after the right end (or the ‘right-flanking nucleotide’) is variable in IS200/605, but is fixed in IS607 (Figure 3B). As the right-flanking nucleotide corresponds to the start of the guide sequence in IS200/605 TnpBs (1), we asked if the same was true for IS607 TnpBs. To test this, we made ISXfa1 reRNA mutants with variable boundaries and performed *E. coli* TAM depletion assays (Figure 3C, Supplementary Figures S4, S7, S9, and Supplementary Text 2). We found that in ISXfa1, the right-flanking nucleotide formed part of the reRNA scaffold, instead of the guide as it would have been for IS200/605 (Figure 3C and Supplementary Figure S4). As such, IS607 TnpBs maintains the same degree of programmability as IS200/605 TnpBs by incorporating an invariable sequence flanking the transposon as part of the RNA scaffold. This suggests that IS607 TnpBs have co-evolved to function with their transposases by adjusting their reRNA boundaries (Supplementary Figures S8, S9, and Supplementary Text 3). This adaptability in the IS607 reRNA scaffold may have been a key feature that enabled the co-option of Fanzor2s by new eukaryotic transposon families.

### A conserved pseudoknot interaction is required for activity of IS607 TnpB

**Figure 4.**
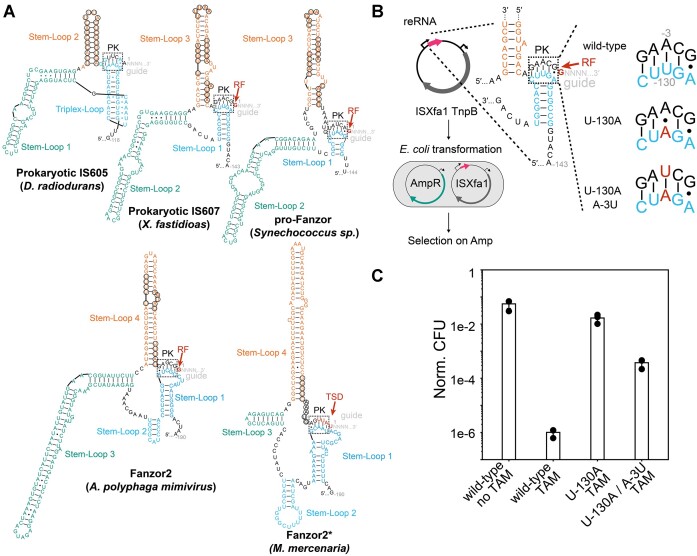
reRNA secondary structures are conserved between TnpBs and Fanzor2s across diverse transposon families. (**A**) Predicted secondary structures of reRNAs associated with various transposons. Right-flanking nucleotides (RF) and target site duplications (TSD) are colored in red. Putative pseudoknot (PK) interactions are highlighted in black dashed boxes. Transposase recognized bases required for transposition are circled in black. (**B**) Experimental design of PK interaction assays. Plasmids containing ISXfa1 TnpB and reRNAs with PK mutations were transformed into *E. coli* harboring an ampicillin resistance (AmpR) plasmid. As ISXfa1 TnpB restricts the AmpR plasmid and leads to ampicillin sensitization, effect of PK mutations was assayed via ampicillin selection. (**C**) Results of the *E. coli* plasmid interference assay confirming the PK interactions. ISXfa1 TnpB activity was disrupted by the U-130A mutation and subsequently rescued by the U-130A/A-3U mutation.

Having defined the boundary of ISXfa1 reRNA scaffold, we next analyzed its reRNA structure. Using RNA-folding analysis (Materials and methods), we found that the ISXfa1 reRNA has three predicted stem-loops and a potential pseudoknot (PK) forming region. This is reminiscent of the previously reported IS200/605 reRNA, whose core structure also involves a PK interaction (Figure 4A) (31,32). To test the functional importance of the putative PK interaction, we generated ISXfa1 reRNA mutants and assayed their activity in an *E. coli* plasmid interference assay. We first destabilized the putative PK interactions by mutating the apical loop in Stem-loop 3 to abrogate a Watson-Crick base pair in the PK (Figure 4B). Compared to the wild-type, the mutant showed substantially reduced activity (<10^−4^ fold) (Figure 4C). Activity was rescued 100-fold by introduction of a second point mutation that restores the Watson-Crick base pair in the PK (Figure 4B and C). The incomplete rescue could be attributed to nucleobase specific contacts between the PK and TnpB, as indicated in the previous ISDra2 TnpB cryo-EM structures (31,32). These results show that a PK interaction is necessary for ISXfa1 TnpB activity. Notably, RNA-folding analysis predicts that this PK structure is be conserved in not only TnpBs, but also Fanzor2s and Fanzor2*s (Figure 4A). This suggests that a PK may be required in TnpBs and Fanzor2s regardless of transposon association.

### Fanzor1 reRNA and protein expression are robust in a native insect host

**Figure 5.**
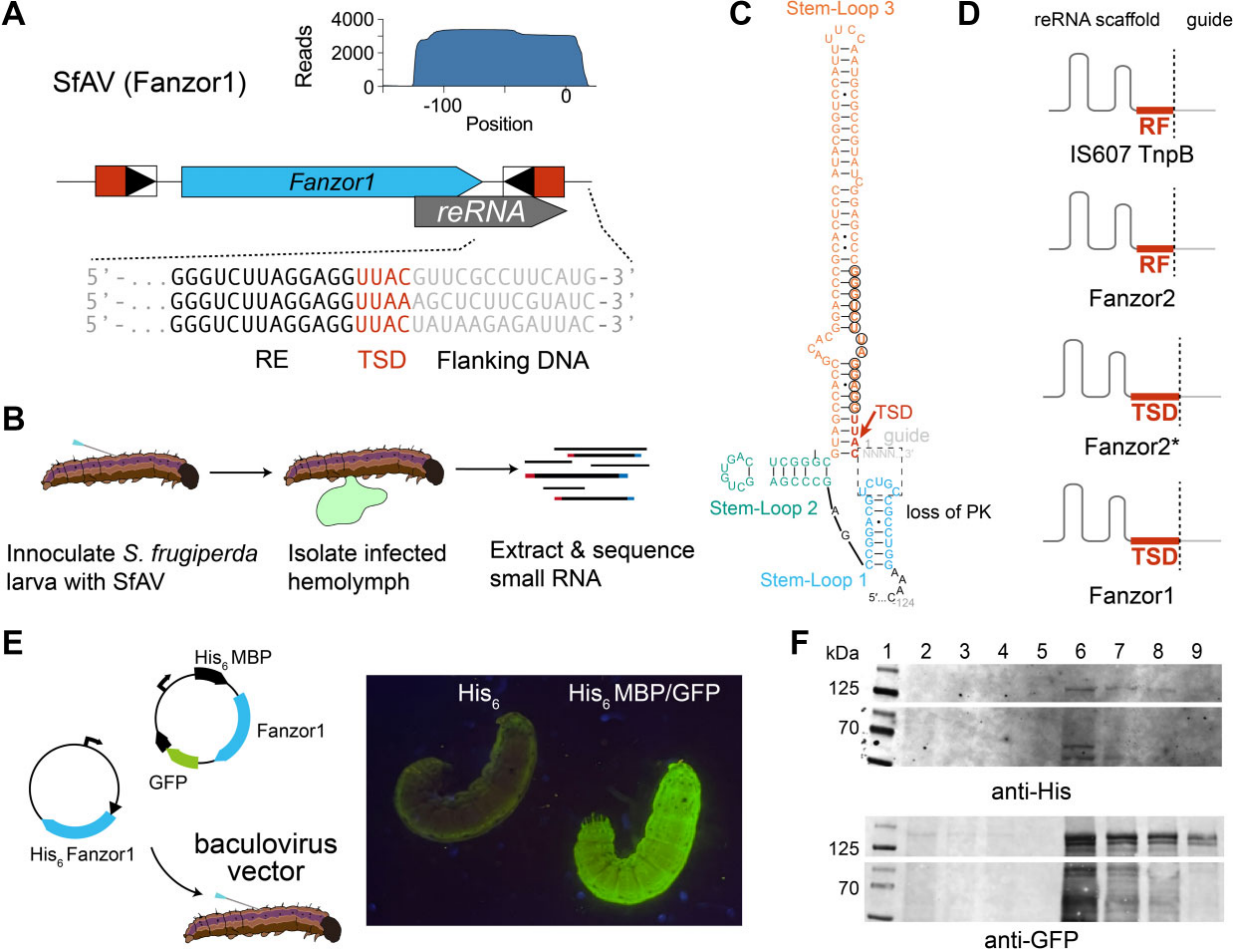
reRNAs of TnpBs and Fanzors use similar strategies to co-function with their transposases. (**A**) Locus organization of Fanzor1 encoding transposon in *S. frugiperda ascovirus 1a* (SfAV). Red box indicates target-site duplication (TSD) which appears as a result of transposition. Boxed triangle indicates terminal inverted repeats (TIR). Inlaid graph shows mapping of small RNA-seq reads onto the reference locus encoding the Fanzor. (**B**) Workflow of *in vivo* Fanzor1 reRNA expression assays using SfAV *and S. frugiperda* larvae. (**C**) Putative secondary structure of SfAV Fanzor1 reRNA. TSD is color coded in red. Region where pseudoknot (PK) was expected is highlighted in black dashed box. Bases corresponding to TIR recognized by the transposase are circled. (**D**) Graphical representation of how boundaries are redefined for reRNAs occurring in different transposon families. Right-flanking nucleotide (RF) in IS607 transposons and TSD in eukaryotic transposons are both incorporated into the reRNA scaffold. (**E**) Schematic depicting MBP-GFP fused SfAV Fanzor1 construct. Picture of *S. frugiperda* larvae infected with recombinant baculovirus encoding his-tagged Fanzor (left) and MBP and GFP tag fused Fanzor is shown (right). (**F**) Western blot confirms expression of SfAV protein *in vivo* using anti-his and anti-GFP primary antibodies. Lane 1: Chameleon ladder. Lanes 2–5: his-tagged Fanzor containing lysate dilutions. Lanes 6–9: MBP and GFP tag fused Fanzor containing lysate dilutions (the full blot is shown in Supplementary Figure S6).

To gain insight into how Fanzors evolve to function in transposons other than IS607 transposons, we investigated the native expression of a Fanzor1 found in a non-IS607 transposon from *Spodoptera frugiperda* ascovirus 1a (SfAV) (8,33,34). The Fanzor-encoding element of SfAV is a non-autonomous transposon present at two copies in the genome that encodes just the Fanzor1 protein (Figure 5A). SfAV Fanzor and related Fanzors from lepidopterans and their viruses appear to be associated with an unknown family of DNA transposon with clear TIRs that cause short TSDs upon insertion (8).

We performed small RNA-sequencing experiments to examine reRNA expression *in vivo* by infecting *S. frugiperda* larvae with SfAV. To do so, we pricked the larvae with an SfAV-contaminated needle to mimic infection by a parasitic wasp, which transmits the virus in nature (Figure 5B). As ascoviruses primarily replicate in the hemolymph, we isolated and sequenced small RNAs from hemolymph samples (Figure 5B) (33). Mapping of small-RNA reads to the SfAV genome revealed that the Fanzor reRNA was one of the most highly transcribed small RNAs (Figure 5A and Supplementary Figure S5). In addition to the SfAV Fanzor, we also examined reRNA expression of two additional insect virus-encoded Fanzor1s in cultured cells using recombinant baculoviruses. In both native and recombinant samples, we found expression of reRNAs that extended ∼15nt beyond the TSD of the transposon (Supplementary Figure S5).

RNA-folding analysis predicted that although PK interactions are absent in Fanzor1 reRNAs, they fold into a three-stem shape whose global architecture is similar to TnpB and Fanzor2 reRNAs (Figure 5C). Notably, the TIRs and the TSDs are predicted to form part of the reRNA scaffold in Fanzor1s (Figure 5C, D, and Supplementary Figure S5), and the same appeared to be true in Fanzor2*s (Figure 4A. The inclusion of the TSD into the reRNA scaffold in Fanzor1s and Fanzor2*s is analogous to the assimilation of the right-flanking nucleotide into the reRNA scaffold in IS607 TnpBs (Figure 5D). This suggests that both Fanzors and IS607 TnpBs overcome sequence constraints imposed by their transposases by recoding them into the reRNA scaffold. This is crucial for Fanzors and TnpBs to maximize their guide programmability, especially given that TSDs of Fanzor1-encoded transposons can reach up to 12 nucleotides in length (8).

We noted that reRNA expression levels *in vivo* using larvae were substantially higher than those observed *in vitro* using cultured Sf9 cells. As SfAV Fanzor protein expression in Sf9 cells resulted in poor yield, we instead expressed the SfAV Fanzor protein *in vivo* using *S. frugiperda* larvae (Figure 5E). Two constructs were tested: a His_6_-tagged SfAV Fanzor protein, and a MBP/GFP-tagged SfAV Fanzor protein. Although we could not detect the His_6_-tagged SfAV Fanzor protein, we observed robust expression of the MBP/GFP-tagged SfAV Fanzor protein *in vivo*, with the larvae turning completely green five days post infection (Figure 5E). Western-blotting with an anti-GFP antibody confirmed the expression of the full-length MBP/GFP-tagged protein (Figure 5F and Supplementary Figure S6). Our results suggest that both Fanzor1 reRNA and protein may be expressed preferentially *in vivo*, implying that Fanzor expression is regulated by additional factors.

### Structural analysis highlights conserved features of the Fanzor lineage

**Figure 6.**
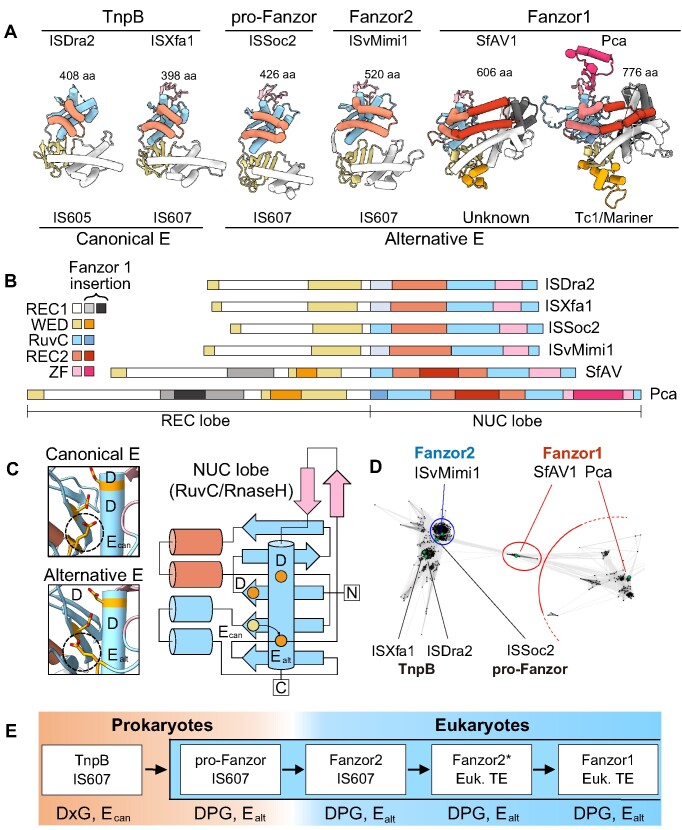
Structural comparisons of TnpBs and Fanzors highlight key conserved features of the Fanzor lineage. (**A**) Example structures of IS200/605 and IS607-associated TnpBs and Fanzors. All models are AlphaFold2 models, except for that of ISDra2 (PDB-ID: 8EXA). Species information: ISDra2, *Deinococcus radiodurans*. ISXfa1, *Xylella fastidiosa*. ISSoc2, *Synechococcus* sp. ISvMimi1, *Acanthamoeba polyphag*a mimivirus. SfAV, *Spodoptera frugiperda* ascovirus 1a. Pca, *Phytophthora cactorum*. (**B**) Schematic representation of the structural organization of TnpBs and Fanzor2s, and regions of novel insertions in Fanzor1s. (**C**) AlphaFold2 structures and protein topology diagram of the two types of RuvC active site organization specific to proteins in (A). Dashed circles in the left, and colored circles in the right panels, indicate the RuvC2 glutamate residue in the canonical E_can_ and the alternative E_can_ locations. (**D**) CLANS analysis for TnpBs and Fanzors sequences. Note that the lepidopteran and viral Fanzors bridge Fanzor2s and the rest of Fanzor1s. (**E**) The proposed evolutionary of Fanzors inferred using extant sequences. Prokaryotic TnpBs with DPG and E_alt_ motifs, or pro-Fanzors, evolved into eukaryotic Fanzor2s via horizontal transfer. Acquisition of Fanzor2s with novel non-IS607 eukaryotic transposons gave rise to Fanzor2*s, one instance of which gave rise to Fanzor1s.

Finally, we compared structural models of Fanzors and TnpBs to determine whether their structural properties support the sequential evolution of Fanzor1s from Fanzors2s and TnpBs (Figure 6A and B). All TnpBs and Fanzors analyzed contained a REC lobe that includes a β-barrel-like wedge domain (WED) and helical REC1 domain, and a NUC lobe that includes the RuvC/RNaseH-fold defined by a β-sheet flanked by double and single α-helices on each side. The double α-helices extend from the NUC lobe to form the REC2 domain, whereas the single α-helix forms part of the nucleolytic pocket bearing the D–E–D catalytic triad. The predicted models of both E_alt_ TnpBs and Fanzors clearly show that the RuvC2 glutamate is placed in a topologically distinct, but consistent location (Figure 6C). Overall, the structural models are consistent with the view that select TnpBs share greater similarities to Fanzors.

Our structural analysis also shows that Fanzor1s are divergent from TnpBs and Fanzor2s. Nevertheless, we see that some structural features are conserved among Fanzor1s. The hallmark structural feature of Fanzor1s is a REC2 domain comprising a pair of kinked α-helices that embraces the RNA/DNA heteroduplex (6). Fanzor1s also feature insertions in the REC1 domain that likely contact the heteroduplex (Figure 6B). These insertions in the REC domains are reminiscent of insertions found in Cas12s thought to enable recognition of longer guide sequences (32). This Fanzor1 adaptation might have enhanced function in eukaryotic genomes whose larger size demands greater specificity.

In addition to REC domain insertions, Fanzor1s contain insertions in the WED domain that alter its β-barrel shape. The WED domain of Fanzor1s contains a characteristic TDG motif, which appears to interact with residues that may interface with the heteroduplex. The WED domain also incorporates a novel α-helix that interacts with the terminal nucleobases of the reRNA scaffold in the previously reported Fanzor1 cryoEM structure (6). Whereas these nucleobases form the PK interaction in reRNAs of TnpBs and Fanzor2s, they constitute a double-stranded RNA stem in reRNAs of Fanzor1s (Figures 4A and 5C). We propose that structural alterations in Fanzor1s that disrupt the ancestral β-barrel architecture in the WED domain facilitated the loss of the PK, alleviating additional sequence constraints on Fanzor1 reRNA.

Structural features particular to and universal among Fanzor1s strongly support their monophyletic origin. Nevertheless, the evolutionary origin of Fanzor1s is less clear compared to Fanzor2s. In our phylogenetic analysis, we found that the clade of Fanzor1s that includes SfAV Fanzor represents an early diverging branch (Figure 2B). Indeed, pairwise similarity analysis of Fanzor sequences independently positions this clade between Fanzor2s and the rest of Fanzor1s (Figure 6D; Materials and methods). The predicted structure of SfAV Fanzor suggests that although proteins in this clade bear the most basic features of Fanzor1s, they lack features found in larger Fanzor1s (Figure 6A, B). Among Fanzor1s, SfAV Fanzor and related Fanzors are structurally most similar to Fanzor2s due to their streamlined architecture. Indeed, SfAV Fanzor is one the most compact Fanzor1s (∼600 residues), and its length is comparable to some Fanzor2s (∼500 residues). In contrast, some Fanzor1s reach up to ∼800 residues in length, largely due to additional insertions in REC1/2, RuvC, and ZF domains (Figure 6A, B). In all, our observations support the view that Fanzor1s initially evolved from a compact ancestor similar to Fanzor2s.

### A model of Fanzor evolution

Based on conserved RuvC signatures, phylogenetic analysis, and association with IS607 TnpAs, the most likely prokaryotic source of Fanzor2s are TnpBs we refer to as pro-Fanzors. Even though the directionality cannot be ascertained (Supplementary Text 1), the striking similarities between pro-Fanzors and Fanzor2s are best explained by cross-domain HGT. Moreover, multiple lines of evidence suggest that Fanzor1s are a monophyletic group that evolved from Fanzor2s. (i) Phylogenetic analysis, (ii) absence of prokaryotic Fanzor1-like proteins, (iii) contemporary evolutionary intermediates we refer to as Fanzor2*s and (iv) conserved structural features are all consistent with this view. Collectively, our findings point toward sequential evolution from pro-Fanzor, to Fanzor2 and finally to Fanzor1.

We propose the following model to explain the evolution of Fanzors from TnpBs. First, pro-Fanzors were horizontally transferred from prokaryotes to eukaryotes or their viruses, generating Fanzor2s (Figure 6E). Fanzor2s were further spread in eukaryotes, often via HGT through viruses, and were adopted by eukaryotic transposons in this process as exemplified by extant Fanzor2*s. Ultimately, a specific group of Fanzor2* gave rise to Fanzor1s that continued to diversify. In this model, pro-Fanzors, Fanzor2s and Fanzor1s all belong to a single lineage of unusual TnpB-family homologs that potentially involved multiple cross-domain HGT events.

## Discussion

Our work provides a plausible explanation for the origin of Fanzors, a widely distributed group of eukaryotic RNA-guided DNA endonucleases. Phylogenetic analysis and structural modeling support the conclusion horizontal gene transfer of a narrow clade of IS607 TnpBs, the pro-Fanzors, gave rise to eukaryotic Fanzors. That all Fanzors evolved from a single prokaryotic source is at great odds with the prevalence of cross-domain horizontal gene transfer and diversity of TnpBs in prokaryotes. This suggests that only a specific and limited group of TnpB-family homologs were suitable for adaptation in eukaryotic systems.

Three properties of pro-Fanzors may explain its successful expansion in eukaryotes. First, pro-Fanzors and Fanzors share a RuvC active site with a repositioned glutamate that may reduce genome-damaging ssDNA *trans*-cleavage activity conserved in other TnpBs and related Cas12 enzymes (6,7,35). Second, pro-Fanzors occur exclusively in cyanobacteria, which have an extensive history of both endosymbiosis with eukaryotes and horizontal gene transfer events including IS607 transposons (9,36,37). Third, pro-Fanzors function together with a double-stranded DNA transposase (IS607 TnpA), as opposed to a single-stranded DNA transposase (IS200/605 TnpA). To our knowledge, there is no evidence of IS200/605 transposons in eukaryotes, suggesting they are not suited for eukaryotic function (9,38,39). Together, these properties of pro-Fanzors may have enabled their cross-domain dissemination and continued divergence as eukaryotic Fanzor2s and the later-evolving Fanzor1s.

The evolutionary relationship between IS607 TnpBs and Fanzors highlights how TnpB-family homologs adapt to function with new transposon families. Notably, we found that the reRNAs encoded in both IS607 and eukaryotic transposons overcome sequence constraints imposed by their transposases in analogous ways. We also found that a putative pseudoknot interaction in the reRNA is conserved among TnpBs and Fanzor2s regardless of their transposon associations. Interestingly, this pseudoknot interaction appears to have been lost in Fanzor1 reRNAs. The absence of pseudoknot-imposed sequence constraints in the reRNA may have facilitated Fanzor1 adoption by a wider range of transposons.

Future studies should investigate why TnpBs are associated only with IS200/605 and IS607 transposons, whereas Fanzors are associated with a large and diverse collection of transposon families. Furthermore, the abundance of some Fanzor-encoding transposons in their host genome, reaching up to hundreds of copies, suggests that Fanzors provide a proliferative advantage to various families of transposons with distinct life-cycles (8). The demonstrated evolutionary success of Fanzors underscores the versatility of RNA-guided endonucleases that are ubiquitously present in all domains of life.

## Supplementary Material

gkad1053_Supplemental_Files

## Data Availability

The data underlying this article are available in the article and in its online supplementary material. Sequencing data underlying this article are also publicly available. small RNA-sequencing data is deposited on GEO under accession GSE246134. Sequencing data from the TAM depletion assays are available on SRA as project ID PRJNA1028567.
