# Supplementary material for: Eukaryotic RNA-guided endonucleases evolved from a unique clade of bacterial enzymes
Source: Nucleic Acids Res. 2023 Nov 16;51(22):12414–27. doi: 10.1093/nar/gkad1053 (PMC10711439; doi:10.1093/nar/gkad1053)
Supplement: gkad1053_Supplemental_Files [file gkad1053_supplemental_files.zip › REVISION_Yoon, Skopintsev, Shi et al_TnpB HGT_manuscript SUPPLEMENTARY INFORMATION.pdf]

Supplementary information

Eukaryotic RNA-guided endonucleases evolved from a unique clade of bacterial enzymes

Peter H. Yoon[1,2*], Petr Skopintsev[2,3*], Honglue Shi[2,4*], LinXing Chen[2,5], Benjamin A. Adler[2,3], Muntathar Al-Shimary[1,2], Rory J. Craig[3], Kenneth J. Loi[1,2], Evan C. DeTurk[2,3], Zheng Li[6], Jasmine Amerasekera[7], Marena Trinidad[2,4], Hunter Nisonoff[8], Kai Chen[1,2], Arushi Lahiri[1,2], Ron Boger[2,3], Steve Jacobsen[6,9], Jillian F. Banfield[2,5], Jennifer A. Doudna[1-4,10-13‡]

[1]Department of Molecular and Cell Biology, University of California, Berkeley; Berkeley, CA, USA; [2]Innovative Genomics Institute; University of California, Berkeley, CA, USA; [3]California Institute for Quantitative Biosciences (QB3), University of California, Berkeley, CA, USA; [4]Howard Hughes Medical Institute, University of California, Berkeley; Berkeley CA, USA; [5]Department of Earth and Planetary Science, University of California, Berkeley, CA, USA; [6]Department of Molecular, Cell and Developmental Biology, University of California, Los Angeles CA, USA; [7]Department of Human Genetics, University of California, Los Angeles CA, USA; [8]Center for Computational Biology, University of California, Berkeley; Berkeley CA USA; [9]Howard Hughes Medical Institute, University of California, Los Angeles CA, USA; [10]Gladstone Institutes; San Francisco, CA, USA; [11]Gladstone-UCSF Institute of Genomic Immunology; San Francisco, CA, USA; [12]Molecular Biophysics and Integrated Bioimaging Division, Lawrence Berkeley National Laboratory; Berkeley, CA, USA; [13]Department of Chemistry, University of California, Berkeley; Berkeley, CA, USA;

*These authors contributed equally to the study
‡Corresponding author. Email: doudna@berkeley.edu

SUPPLEMENTARY TEXT

1. *Sequence features and phylogenetics of pro-Fanzors from cyanobacteria*

Our phylogenetic analysis suggests that IS607 TnpBs primarily found in cyanobacteria (pro-Fanzors) are most closely related to Fanzor2s. Indeed, pro-Fanzors display features that would be expected of an ancestral Fanzor. Like both Fanzor1s and Fanzor2s, these TnpBs have a DPG motif at RuvC1, and a repositioned RuvC2 E-residue (E_{alt}). Moreover, like Fanzor2s, these TnpBs are encoded in IS607 elements, but not IS200/605 elements. Consistent with their close phylogenetic relationship with Fanzors, pro-Fanzors share great sequence similarity to Fanzor2s. The only feature we can discern between pro-Fanzors and Fanzors is that pro-Fanzors tend to be shorter because they lack the disordered nuclear localization found in the N-term of Fanzor2s (1, 2).

Although our analysis provides strong support for grouping pro-Fanzors and Fanzor2s into a single clade, we are currently unable to infer details regarding the crossdomain HGT event(s). Of note, the topology of the phylogenetic tree that includes pro-Fanzors, Fanzor2s, and Fanzor1s shows that pro-Fanzors form a distinct subclade that is nested deeply within the Fanzor2 clade. This may either result from the Fanzor2 clade being the product of multiple HGT events involving pro-Fanzors, or pro-Fanzors actually arising from HGT of Fanzor2s. Even though we cannot rule out the possibility of the latter, pro-Fanzors are found broadly in cyanobacteria, which is consistent with them being ancient in prokaryotes.

2. *Determining the reRNA boundary of IS607 TnpB*

Insertion sequence analysis revealed that IS607 TnpAs insert transposons via recombination of matching a GG dinucleotide motif. As discussed in the main text, the first nucleotide of the transposon right-flank is fixed as G. This raises the question of whether the right-flank G is in the programmable guide region or in the reRNA scaffold for IS607 TnpB. To precisely determine the reRNA boundary, we deleted bases in this region in the TAM depletion assay. If the right-flank G is in the programmable guide region, the IS607 TnpB will still be active even after deleting two G bases (Supplementary Figure S4). In contrast, if the right-flank G is in the reRNA scaffold, the IS607 TnpB will lose function after removing two G bases (Supplementary Figure S4). To test which model is correct, we deleted two G bases in this region and replicated the TAM depletion assays. We observed that deletion of two G's leads to no TAM signal in the TAM weblogo suggesting loss of function of IS607 TnpB (Figure 3C and Supplementary Figure S4). These results suggest that in contrast to IS200/605 transposons, the first nucleotide of the right flanking sequence in IS607 elements is part of the reRNA scaffold rather than the guide. This architecture ensures that the same degree of reprogrammability is maintained in IS607 TnpBs, despite having a fixed nucleotide flanking the transposon. Further *E.coli* plasmid interference assay also confirms that IS607 TnpB functions as an RNA-guided endonuclease activity (Supplementary Figure S7).

3. *IS607 TnpBs co-evolved to function with their transposases in the transposon life cycle*

As discussed in the main text, IS607 TnpBs co-evolved with their transposases by modifying their reRNA boundaries. The co-evolution of IS607 TnpBs with their TnpAs suggests that IS607 TnpBs are also involved in the transposon life cycle, as observed for IS200/605 TnpBs. In IS200/605 transposons, the insertion specificity of the TnpA perfectly matches the TAM of the TnpB, which results in empty site double-stranded break by TnpB and reconstitution of the lost transposon by DNA repair (3–5). We therefore investigated whether the same was true in IS607 elements.

ISXfa1 TnpA likely recognizes and mediates transposition into SGG motifs (where S is C or G), as a closely related IS607 element (>80% sequence identity) catalyzes insertion into SGG motifs (6, 7). In contrast to this, our TAM depletion assays suggested that ISXfa1 TnpB strongly prefers a GGG TAM. To confirm this preference, we used an *E. coli* plasmid interference assay to evaluate ISXfa1 TnpB's ability to cleave DNA targets bearing either a GGG or CGG motif (Supplementary Figure S8A). We found that ISXfa1 TnpB mediates robust interference of target plasmids containing a GGG TAM, but shows no activity against those containing a CGG TAM (Supplementary Figure S8A). This suggested that ISXfa1 TnpB strictly recognizes a GGG TAM, implying that the TnpA insertion specificity may be more promiscuous than the TnpB TAM in IS607 elements.

A recent study has shown that TnpBs help ensure the persistence of their transposons by creating double-stranded breaks at sites that have lost the transposons (5). TnpBs accomplish this by recognizing a TAM and target sequence that is disrupted by the transposon insertion, but reconstituted by transposon excision. In instances where the transposon is inserted into a site that does not match the TnpB TAM, we can expect that the TnpB would be unable to serve its function. Presumably, these insertion events would be selected against the insertion events that allow the TnpB to function. Consistent with this, we observe that the vast majority of IS607 copies in *Xylella fastidiosa* and *Helicobacter pylori* are found inserted into GGG sequences, despite the SGG insertion specificity of the TnpA (Supplementary Figure S8B).

Collectively, our observations suggest that in IS607 elements, TnpBs influence the insertion profile of the transposon by selecting for TAM bearing insertion sites following promiscuous insertion by the TnpA. The mismatch TnpA and TnpB sequence specificities in IS607 elements is likely caused by the distinct transposition mechanism of IS607 TnpAs that is unusual among recombinases. Note that this mechanism could be conserved in not only IS607 encoded Fanzors, but also Fanzor1s encoded in diverse transposons since many Fanzor associated transposases are known to have little sequence specificity.

SUPPLEMENTARY REFERENCES

1. Jiang,K., Lim,J., Sgrizzi,S., Trinh,M., Kayabolen,A., Yutin,N., Bao,W., Kato,K., Koonin,E.V., Gootenberg,J.S., *et al.* (2023) Programmable RNA-guided DNA endonucleases are widespread in eukaryotes and their viruses. *Sci. Adv.*, **9**, eadk0171.
2. Saito,M., Xu,P., Faure,G., Maguire,S., Kannan,S., Altae-Tran,H., Vo,S., Desimone,A., Macrae,R.K. and Zhang,F. (2023) Fanzor is a eukaryotic programmable RNA-guided endonuclease. *Nature*, 10.1038/s41586-023-06356-2.
3. Karvelis,T., Druteika,G., Bigelyte,G., Budre,K., Zedaveinyte,R., Silanskas,A., Kazlauskas,D., Venclovas,Č. and Siksnys,V. (2021) Transposon-associated TnpB is a programmable RNA-guided DNA endonuclease. *Nature*, **599**, 692–696.
4. Xiang,G., Li,Y., Sun,J., Huo,Y., Cao,S., Cao,Y., Guo,Y., Yang,L., Cai,Y., Zhang,Y.E., *et al.* (2023) Evolutionary mining and functional characterization of TnpB nucleases identify efficient miniature genome editors. *Nat. Biotechnol.*, 10.1038/s41587-023-01857-x.
5. Meers,C., Le,H.C., Pesari,S.R., Hoffmann,F.T., Walker,M.W.G., Gezelle,J., Tang,S. and Sternberg,S.H. (2023) Transposon-encoded nucleases use guide RNAs to promote their selfish spread. *Nature*, 10.1038/s41586-023-06597-1.
6. Chen,W., Mandali,S., Hancock,S.P., Kumar,P., Collazo,M., Cascio,D. and Johnson,R.C. (2018) Multiple serine transposase dimers assemble the transposon-end synaptic complex during IS607-family transposition. *eLife*, **7**, e39611.
7. Kersulyte,D., Mukhopadhyay,A.K., Shirai,M., Nakazawa,T. and Berg,D.E. (2000) Functional Organization and Insertion Specificity of IS607, a Chimeric Element of Helicobacter

pylori. *J. Bacteriol.*, **182**, 5300–5308.

SUPPLEMENTARY FIGURES

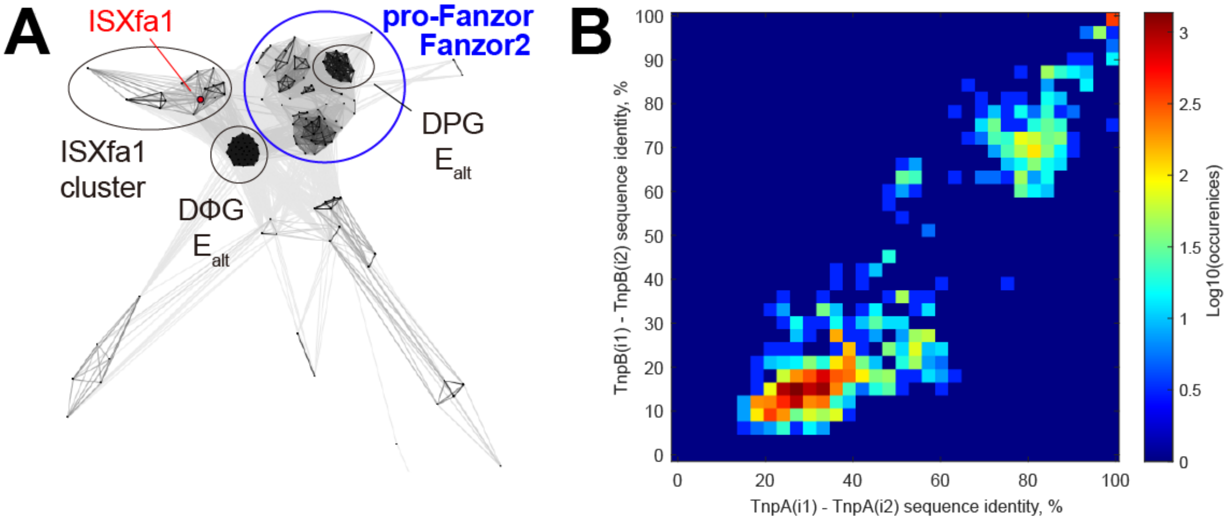

Supplementary Figure S1. Co-conservation analysis of TnpA and TnpB/Fanzor pairs.

A) CLANS clustering visualization of IS607-associated TnpBs from the ISFinder database, TnpBs with C-terminally shifted glutamate and DΦG motifs, and Fanzor2s. Only the TnpBs and Fanzors from the circled clusters were picked for further analysis in (B).

B) Figure shows a sequence identity histogram of pairs of TnpA and TnpB/pro-Fanzor/Fanzor2 between two distinct IS607 elements (i1 and i2) in the Fanzors-TnpB clusters. The calculated Pearson correlation coefficient 0.95 indicates high similarity of IS607 transposable elements within the selected clusters.

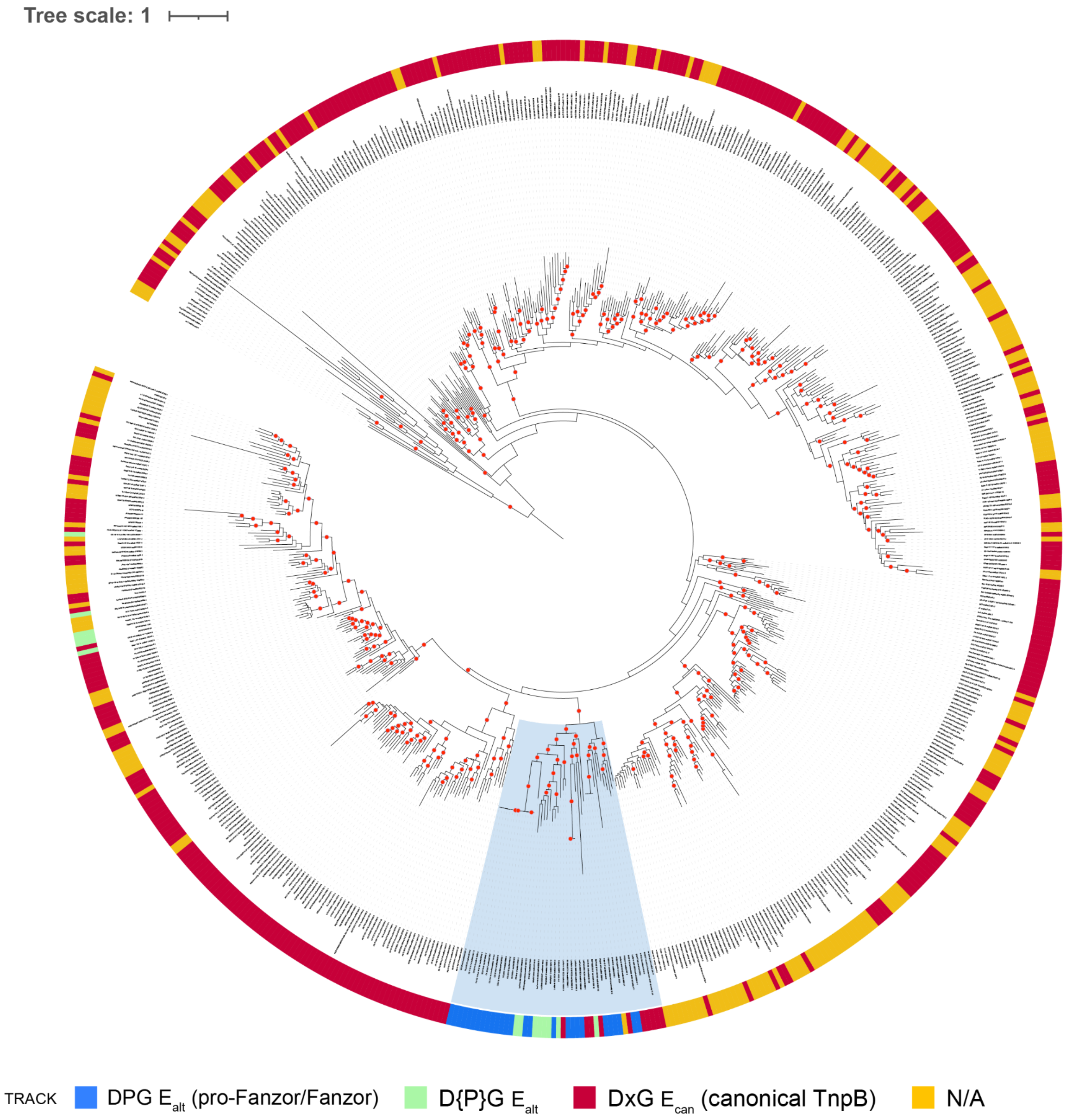

Supplementary Figure S2. Phylogenetic tree of metagenomics derived IS607 TnpAs annotated based on their TnpB partners.
Red dot indicates >95 UF-bootstrap values. Light blue range indicates clade containing TnpAs whose TnpB partner is a pro-Fanzor or a Fanzor. Track annotates associated TnpB as defined by RegEx shown in Supplementary Table S1 as either sharing RuvC1 and RuvC2 signatures (blue), or just RuvC2 signature (green) with Fanzors. Red indicates that the associated TnpB has a typical TnpB architecture containing the non-shifted RuvC2 residue and definitive ZF motifs as captured by the RegEx, whereas red indicates that the associated TnpB was not captured by any of the RegEx. This tree was rooted using Tn3 resolvase, which is a distant homolog of IS607 TnpA, as an outgroup.

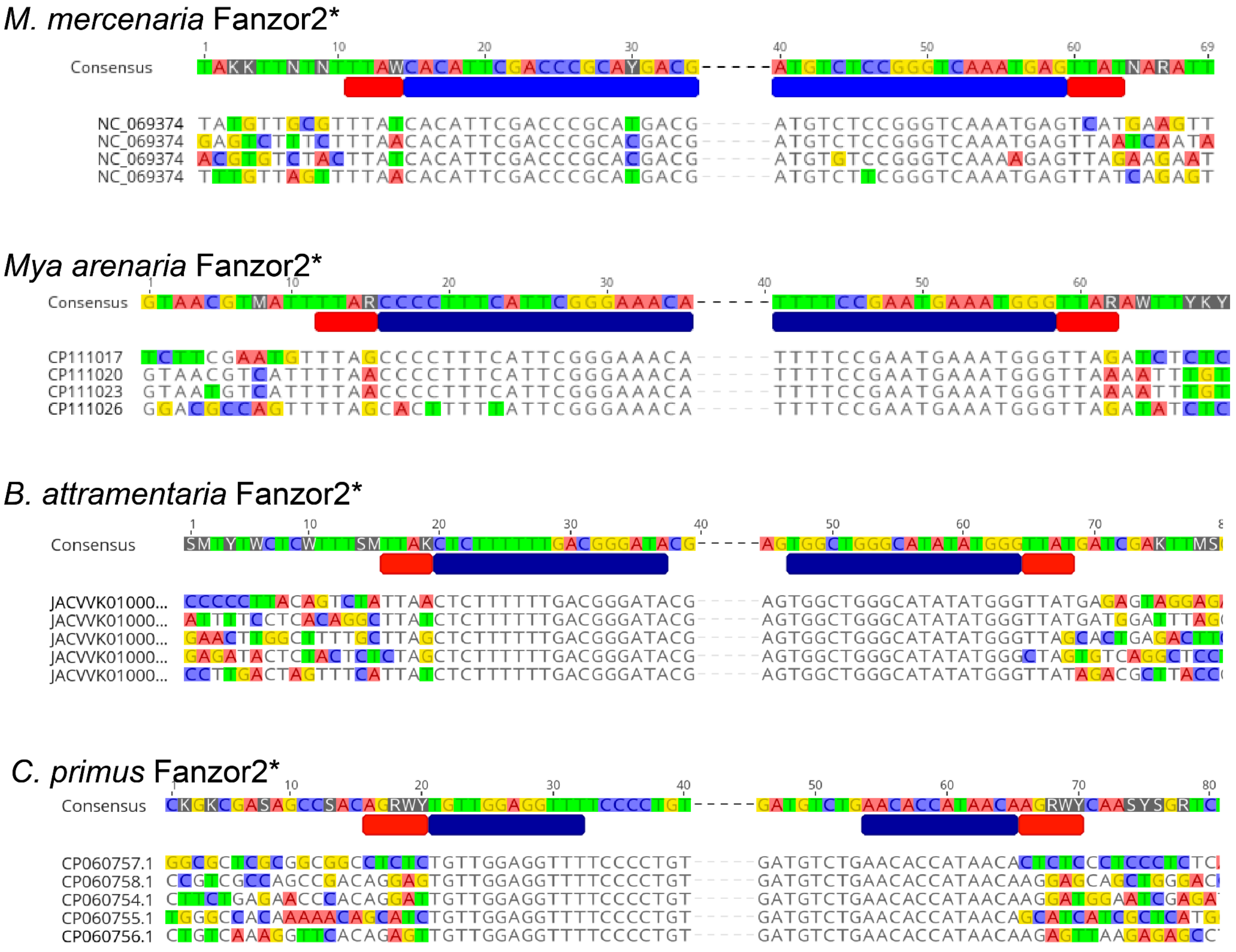

Supplementary Figure S3. Analysis of Fanzor2 encoded transposon signatures reveal Fanzor2 capture by non-IS607 transposons in eukaryotes.
Red bars indicate regions corresponding to target site duplication (TSD). Blue bars indicate regions corresponding to terminal inverted repeats, which appeared to be degenerate in some instances.

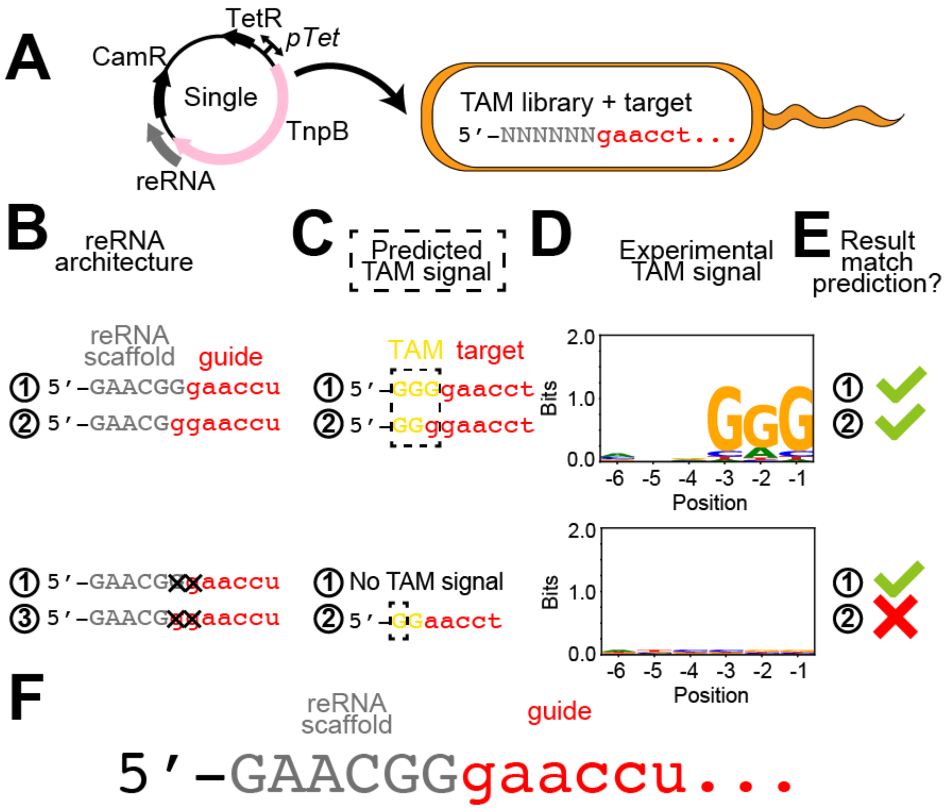

Supplementary Figure S4. Determining the IS607 (ISXfa1) reRNA boundary of the scaffold and guide.

A) Cartoon showing the scheme of *E.coli* TAM depletion assay. The pSingle is transformed into a competent cell harboring a plasmid encoding a 6N TAM library flanking a target site. Recognition of the TAM and target site by TnpB will result in depletion of cell population bearing the TAM sequences.

B) Two hypothesized ISXfa1 reRNA boundaries of the scaffold and guide . For boundary ①, the 3′ of the scaffold ends with GAACGG, for boundary ②, the 3′ of the scaffold ends with GAACG, and for boundary. Deleting two junctional “G” in between the scaffold and guide will result in different TAM signals for different hypothesized boundaries.

C) Predicted TAM signal given different hypothesized boundaries and different deletion mutations. The TAM signals are highlighted in black box.

D) Experimental TAM motif.

E) Whether experimental results match the prediction.

F) Confirmed ISXfa1 reRNA boundary of the scaffold (gray) and guide (red).

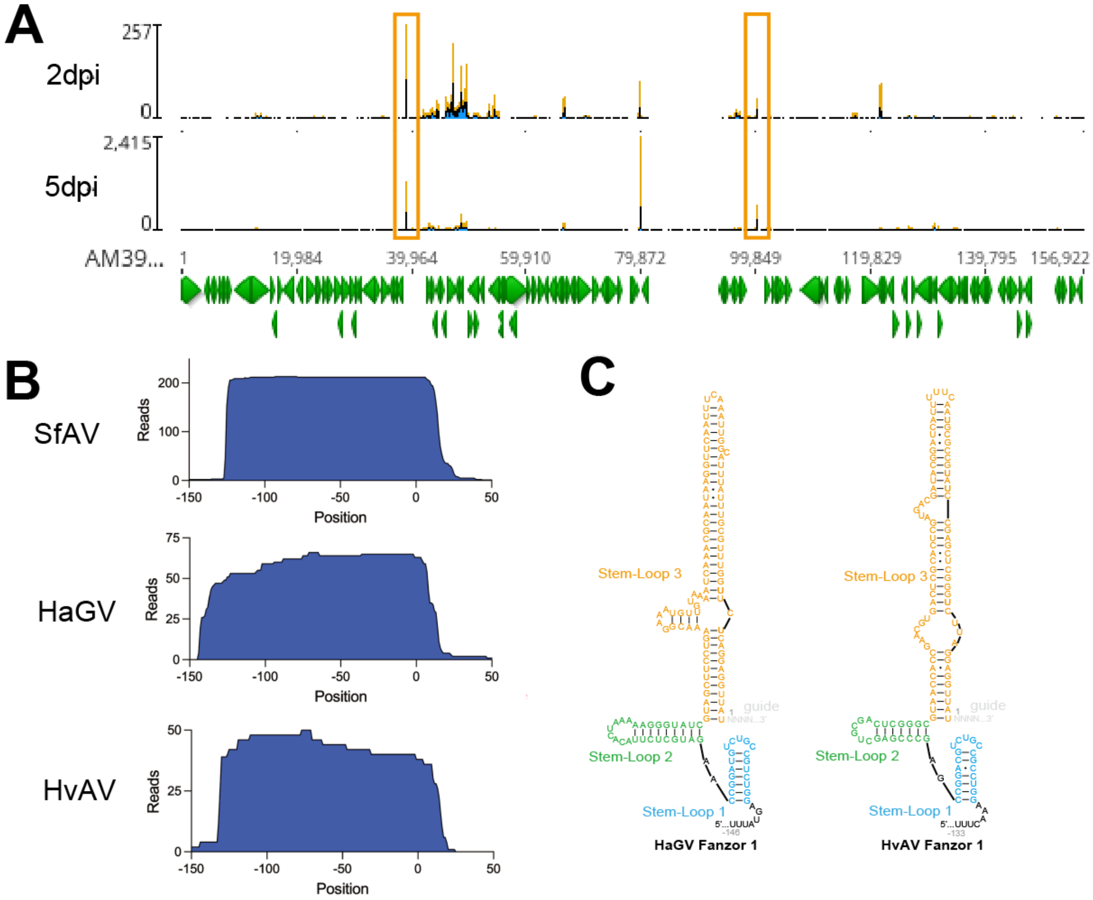

Supplementary Figure S5. reRNA expression from insect virus Fanzors.

A) Mapping of small RNA (<200 nt) reads from the *in vivo* expression experiment to the SfAV genome. reRNA for SfAV Fanzor is one of most highly expressed transcripts, and increased overtime from 2dpi to 5dpi.
B) Mapping of small RNA reads from Expi-Sf9 cells using recombinant baculovirus to the artificial transposon encoding locus.
C) Predicted HAgv and HVav Fanzor1 reRNA secondary structures.

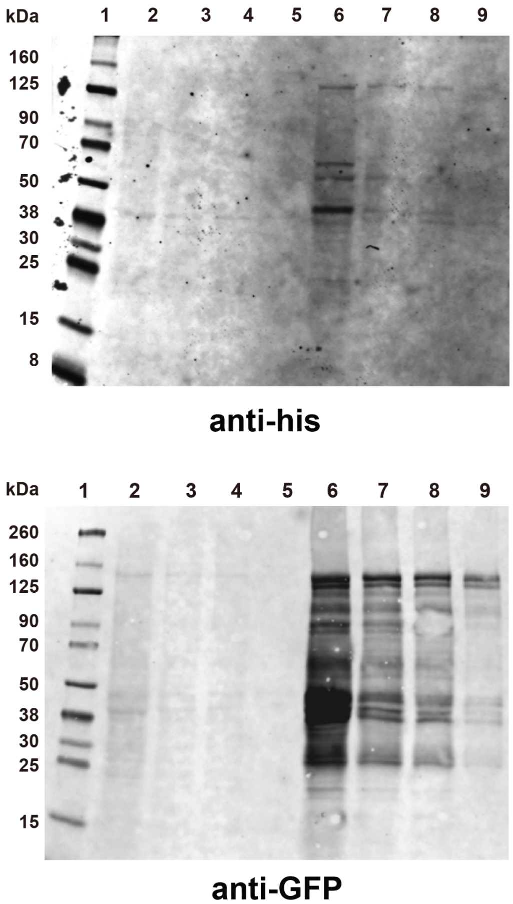

Supplementary Figure S6. Full western blot images shown in Figure 5.
Lysate from *in vivo* expression was loaded in 5x serial dilutions. Top: anti-his primary antibody. Bottom: anti-GFP primary antibody.

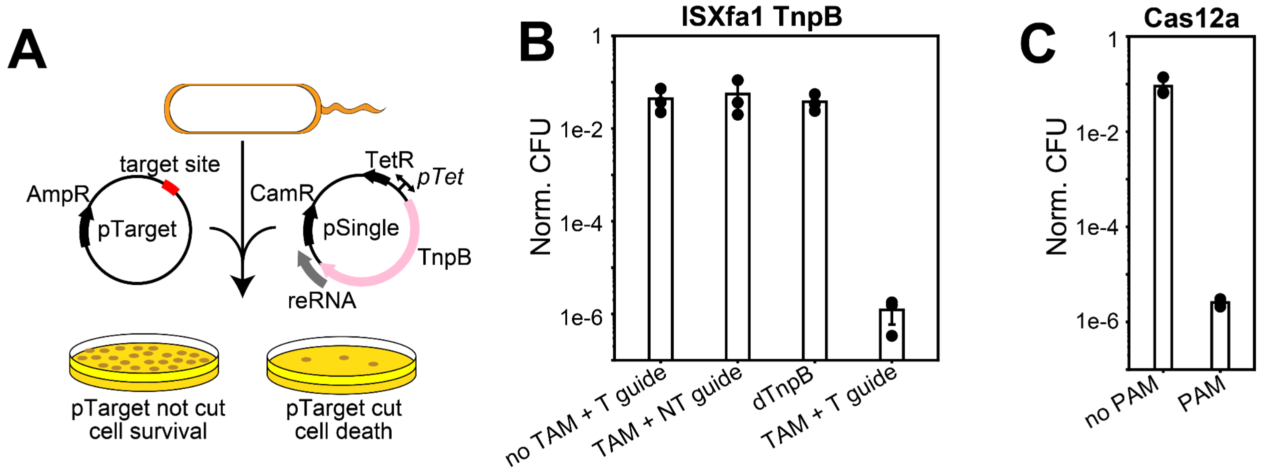

Supplementary Figure S7. IS607 (ISXfa1) TnpB plasmid interference assays in *E. coli*.

A) Cartoon diagram depicting workflow and construct design of *E. coli* plasmid interference assay
B) Quantification of E. coli plasmid interference assay results for ISXfa1 TnpB demonstrating reRNA, TAM, and RuvC active site dependence.
C) Positive control quantifying *E. coli* plasmid interference assay results for LbCas12a, demonstrating comparable activity with ISXfa1 TnpB.

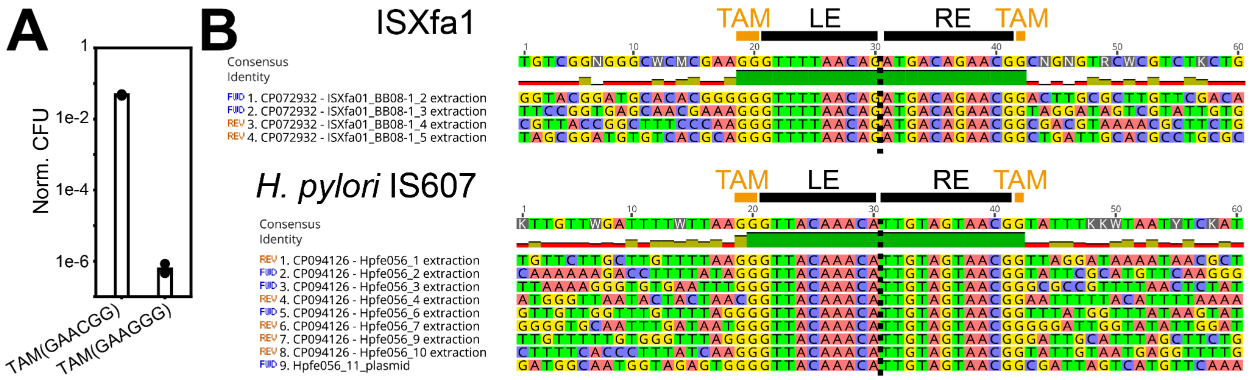

Supplementary Figure S8. IS607 TnpB strictly recognizes the "GGG" TAM and also influences the insertion profile of the transposon.

A) Quantification of E. coli plasmid interference assay showing that ISXfa1 TnpB effectively targets a GGG TAM, but not a CGG TAM.

B) MSA of IS607 elements in *Xylella fastidiosa* (ISXfa1) and *Helicobacter pylori* (*H. Pylori* IS607) genomes and plasmids demonstrating conservation of GGG sequences at the insertion site.

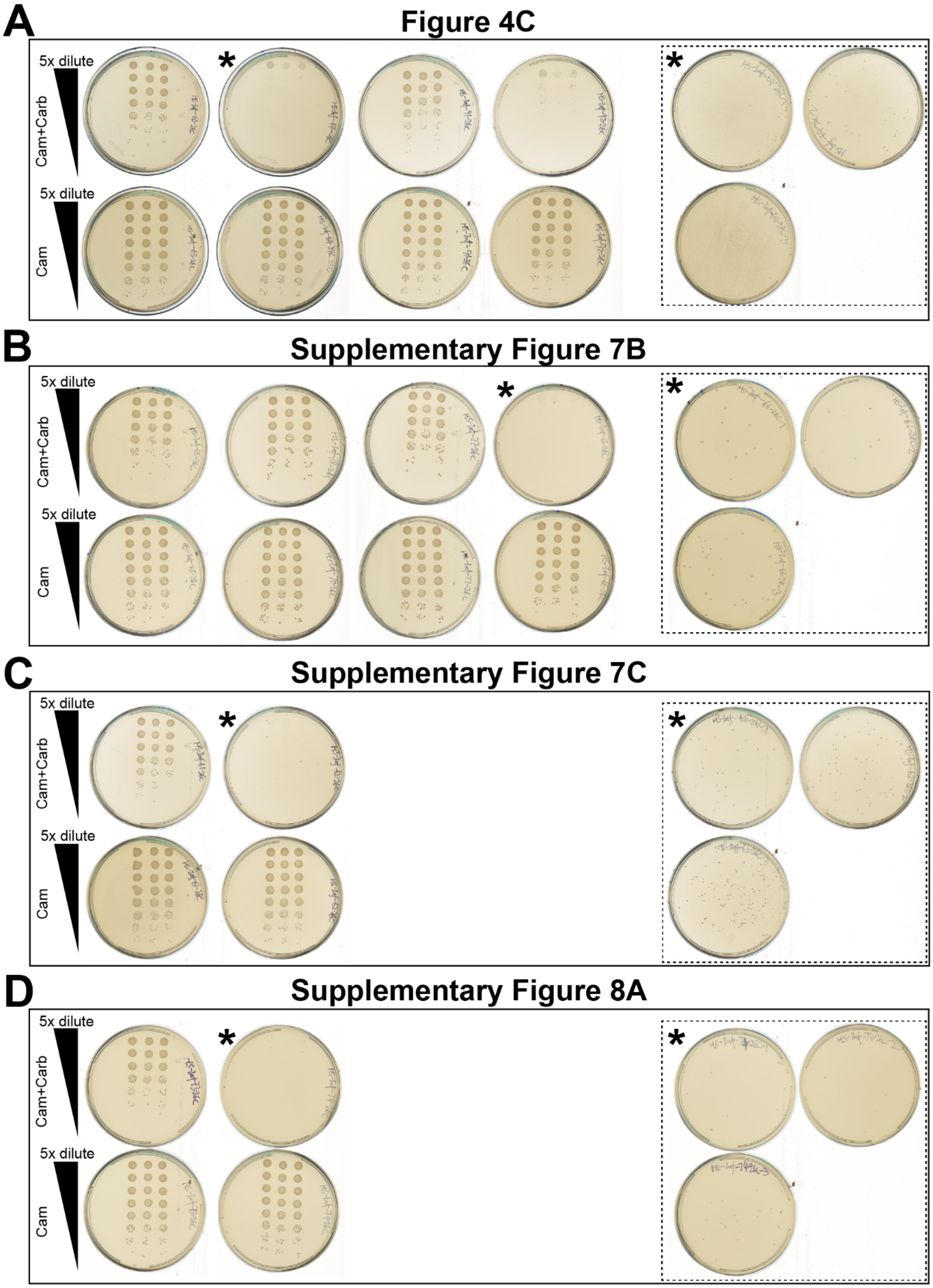

Supplementary Figure S9. The plate images of E.coli plasmid interference assay.
5 μL of the 5-fold serial dilution of the 1mL recovery culture after double-transformation were plated on double antibiotic LB-Agar plates (upper row) and single antibiotic plate (lower row). Each plate contains triplicate transformations. For plates with no visible colonies (asterisk), 400

μL of the original 1 mL recovery culture were plated on the double antibiotic plate (dash insets). (A) corresponds to Figure 4C, (B) to Supplementary S7B, (C) to Supplementary Figure S7C (D) to Supplementary S8A.

SUPPLEMENTARY TABLES

All the supplementary tables are provided in an Excel spreadsheet "yoon_et_al_NAR_2023_supplementary_tables.xlsx".

Supplementary Table S1. Representatives of TnpB-family homologs identified from the ggKBase.

Supplementary Table S2. Fanzor HMM hits identified from NCBI.

Supplementary Table S3. Curated list of structurally complete Fanzor sequences

Supplementary Table S4. Summary of contigs containing alleged Fanzor sequences with canonical RuvC2 organization.

Supplementary Table S5. TnpB/Fanzors with alternative RuvC2 organization.

Supplementary Table S6. Summary of contigs containing Fanzor1-like proteins in prokaryotes.

Supplementary Table S7. Regular Expression used to categorize TnpB sequences
